# Fault stress inversion reveals seismogenic asperity of the 2011 Mw 9.0 Tohoku-Oki earthquake

**DOI:** 10.1038/s41598-019-47992-x

**Published:** 2019-08-19

**Authors:** Zhoumin Xie, Yongen Cai, Chi-yuen Wang, Shoichi Yoshioka, Momo Tanaka

**Affiliations:** 1 Institute of Crustal Dynamics, China Earthquake Administration, Beijing, 100085 China; 2 Department of Earth, Planetary, and Space Sciences, University of California, Los Angeles, CA 90095-156702 USA; 3 Institute of Theoretical and Applied Geophysics, School of Earth and Space Sciences, Peking University, Beijing, 100871 China; 4 Department of Earth and Planetary Science, University of California, Berkeley, CA 94720 USA; 5 Research Center for Urban Safety and Security, Kobe University, Kobe, 657-8501 Japan; 6 Department of Planetology, Graduate School of Science, Kobe University, Kobe, 657-8501 Japan

**Keywords:** Seismology, Applied mathematics

## Abstract

We predict, with a model (earthquake stress model) that inverts the displacements documented at 163 GNSS onshore stations of the GEONET, the change of shear and normal stresses on the megathrust near the Japan Trench over the seven years before the 2011 Mw 9.0 Tohoku-Oki earthquake. We find three areas on the megathrust with greater accumulations of shear and normal stresses before the earthquake, which match the ruptured areas of the mainshock and two largest aftershocks (*M*_w_ 7.8 and 7.4) that occurred within half an hour after the mainshock. We also find that the change of normal stress on the fault before the earthquake is not uniform but increases in the up-dip portion (shallower depth) of the fault from the hypocenter and decreases in the down-dip portion. We infer that the occurrence of the giant earthquake at the shallow portion of the megathrust may be attributed to the increase of the normal stress there, which leads to an increase of fault shear strength and allows more elastic strain energy to accumulate to prepare for the next big earthquake. Based on these results we propose a new concept of the seismogenic asperity as the area of greater accumulations of shear and normal stresses. The method presented here may be useful for predicting the rupture zone of future large earthquakes.

## Introduction

Many attempts have been made to identify the location of the seismogenic zones of oncoming earthquakes. The concept of seismic gap^1^ on plate boundaries is based on the distribution of seismicity and the gap is considered as possible sites of future large earthquake. It has been used in long-term forecasting of the possible locations and magnitude of future large earthquakes. The major plate boundaries are classified into six categories of seismic potential gaps for large earthquakes^2^. The mere presence of seismic gaps is not sufficient for predicting future earthquakes was pointed out because some seismic gaps may slip aseismically^3^. Furthermore, multiple gaps may be present on the same plate boundary, and it is difficult to predict which gap may break in the near future. The state of interseismic locking of subduction megathrusts is commonly used to predict seismogenic zones and has been modeled with the back-slip method^4^. Using this method, a number of interseismic locking models based on an elastic Earth model were constructed prior to the 2011 *M*_w_ 9.0 Tohoku-Oki earthquake at the Japan Trench by inverting geodetic observations and predicted significant interseismic slip deficits down to depths exceeding 40 km, with some extending as deep as ~100 km^5–8^. The slip deficits in the depth range of 10–50 km, peaking around 35 km were also predicted using a simplified, layered viscoelastic model^9^. All the predicted slip deficits centered in fault areas landward of, and thus significantly deeper than, the main rupture area of the 2011 *M*_w_ 9.0 Tohoku-Oki earthquake, and they all predicted interseismic creep in the area ruptured by the 2011 earthquake near the trench^10^.

After the Mw 9.0 Tohoku-Oki earthquake, the spatial correlation of interseismic coupling and coseismic rupture extent of the earthquake are studied by inverting GPS data^11^; the inferred locked asperities are about 25% the size of the rupture areas of the mainshocks. The interseismic GPS data are fitted better^12^ if the assumption of zero interseismic coupling along the Japan trench is removed.

The systematic mismatch of the model-predicted locked asperities and the actual rupture zone of the 2011 earthquake may be due to the shortcomings of the kinematic models used in the inversion for the fault slip-deficit; these may include the lack of consideration of the effect of normal stress on the fault, topography, heterogeneity and anisotropy of the earth media^10^.

**Figure 1:**
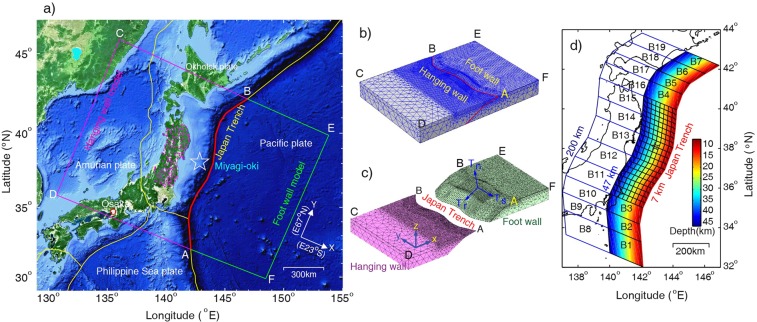
(**a**) Map of Japan and the Japan Trench (Map data: Google, DigitalGlobe), showing the study region of the 2011 *M*_w_ 9.0 Tohoku-Oki earthquake in the rectangle outlined by magenta lines in the North American plate and green lines in the Pacific plate. The GPS stations onshore (from http://www.gsi.go.jp/ENGLISH/page_e30233.html) and the epicenter (from http://www.jma.go.jp) are marked, respectively, by magenta points and a star symbol. (**b**) Earthquake stress model based on the elastic theory and finite method, the fault marked by red line is simplified as an interface across which normal displacement is continuous and the stress vectors on its two sides have the same magnitude but are opposite in direction. The dimension of the finite element model: 1700 km and 1200 km in the directions normal and parallel to the trench, respectively, and 200 km in the vertical. The fault trace (i.e., the Japan Trench) is marked by a red line. (**c**) The hanging wall and footwall of the earthquake fault. The three components of the stress accumulation on the fault boundary, T_*r*_, T_*s*_ and T_*n*_, are only shown on the footwall for clarity. The subscripts *r* and *n* denote the directions along and normal to the subduction direction; the subscript *s* denotes the direction along the fault strike. Displacements on the lateral boundaries and bottom of the model are supported by rollers and are free on the surface. (**d**) Sub-faults on fault surface. Large sub-faults marked by ‘B’ are far from the mainshock hypocenter; small ones are near the hypocenter. Each small sub-fault is 25 km × 25 km in dimension (modified from *Xie and Cai*, 2018).

An inversion method based on an earthquake stress model (Methods) was proposed^13^ for determining not only the shear stress change on fault surface but also the normal stress change on the ruptured fault, by which they directly determined the coseismic stress drop of the 2011 M_w_ 9.0 Tohoku-Oki earthquake from inverting the coseismic GNSS displacements. In this study we use the same method that inverts the displacements (see Table S1) at 163 GPS onshore stations of the GEONET in the period from 2004 to 2011 to determine stress accumulation on the megathrust over a period of seven years *prior* to the earthquake. Figure 1a shows the study region and the earthquake stress model based on the finite element method. The finite element model consists of two elastic tectonic blocks in contact along an interface representing the subduction megathrust that hosts the future earthquake (Fig. 1b,c) and is used in inverting the GNSS displacements to determine the stress changes during the seven years before the 2011 M_w_ 9.0 Tohoku-Oki earthquake. We find from the inversion three areas of greater accumulations of shear and normal stresses on the megathrust, which are consistent with the location of the Mw 9.0 Tohoku-Oki earthquake and the two largest aftershocks south and north to the mainshock. Using the simulated change of normal stress on the megathrust, we explain why the Tohoku earthquake occurred at the shallow portion of the megathrust and why the predicted locking areas by the back-slip models missed the rupture area of the mainshock. Based on these results we propose a new concept of the seismogenic asperity as the area of greater accumulations of shear and normal stresses. The method used in this study may be useful for locating the potential rupture zones of future large earthquakes.

## Results

**Figure 2:**
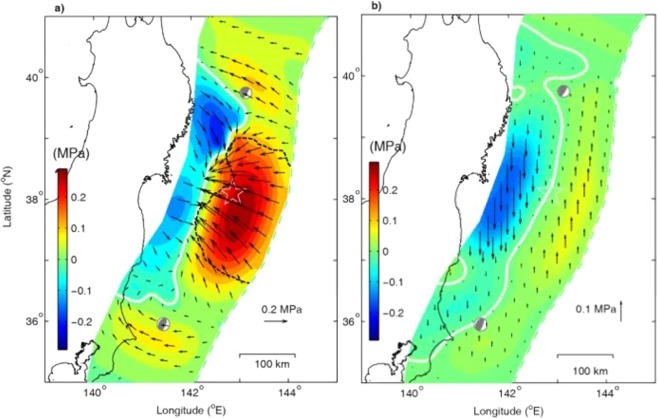
(**a**) Inverted shear stress and (**b**) normal-stress accumulations on the fault surfaces of the hanging wall, respectively, prior to of the Tohoku-Oki earthquake. The direction and length of the arrow represent the direction and magnitude of shear or normal stresses, green dot line marks the rupture termination boundary of the mainshock, determined by the contour of zero-shear stress change^14^. Compression is positive. The epicenter of the 2011 *M*_w_ 9.0 Tohoku-Oki earthquake is marked by a star symbol. Beach balls in the north-east and south-west of the show focal mechanisms of two aftershocks with *M*_w_ 7.4 and *M*_w_ 7.8 of the Tohoku-Oki earthquake, occurring at UTC 06:08 and 06:15 on March 11, respectively.

Figure 2a,b show the fault stresses determined by the inversion in the region from the trench to ~45 km depth, approximately the downdip limit of interplate earthquakes^14,15^. Smaller stresses outside this region are listed in Table S2 but not graphically displayed for the sake of clarity. Figure 2a shows that three prominent zones of high shear-stress accumulation (positive for stress increase) occur near the trench, each centers about 15 km below the seafloor and is bounded by a contour of zero-shear stress. The zone of the fastest shear-stress accumulation, amounting to 0.3 MPa over the seven years, hosts the hypocenter and main rupture zone of the future *M*_w_ 9.0 Tohoku-Oki earthquake^13^. The normal stress also increases in this zone over the seven years (Fig. 2b), with a maximum of ~0.07 MPa. Two minor zones of shear and normal stress accumulation occur to the south and the north of the main rupture zone (Fig. 2), with maximal shear stress of ~0.1 MPa and ~0.03 MPa, respectively, over the seven years and match the hypocenters of the two largest aftershocks with *M*_w_ 7.8 and 7.4^16^.

The inversion also shows the areas of decreases of the shear and normal stresses, bounded by contours of zero-shear and zero-normal stresses (Fig. 2a,b), respectively. The area of shear stress decrease occurs between latitudes 36°N and 40°N, down-dip of the main area of the shear stress accumulation, with a maximum decrease of ~0.14 MPa, to the north of the mainshock hypocenter. The area of normal stress decrease occurs down-dip of the main area of the normal stress accumulation, with a maximum decrease of ~0.15 MPa.

**Figure 3:**
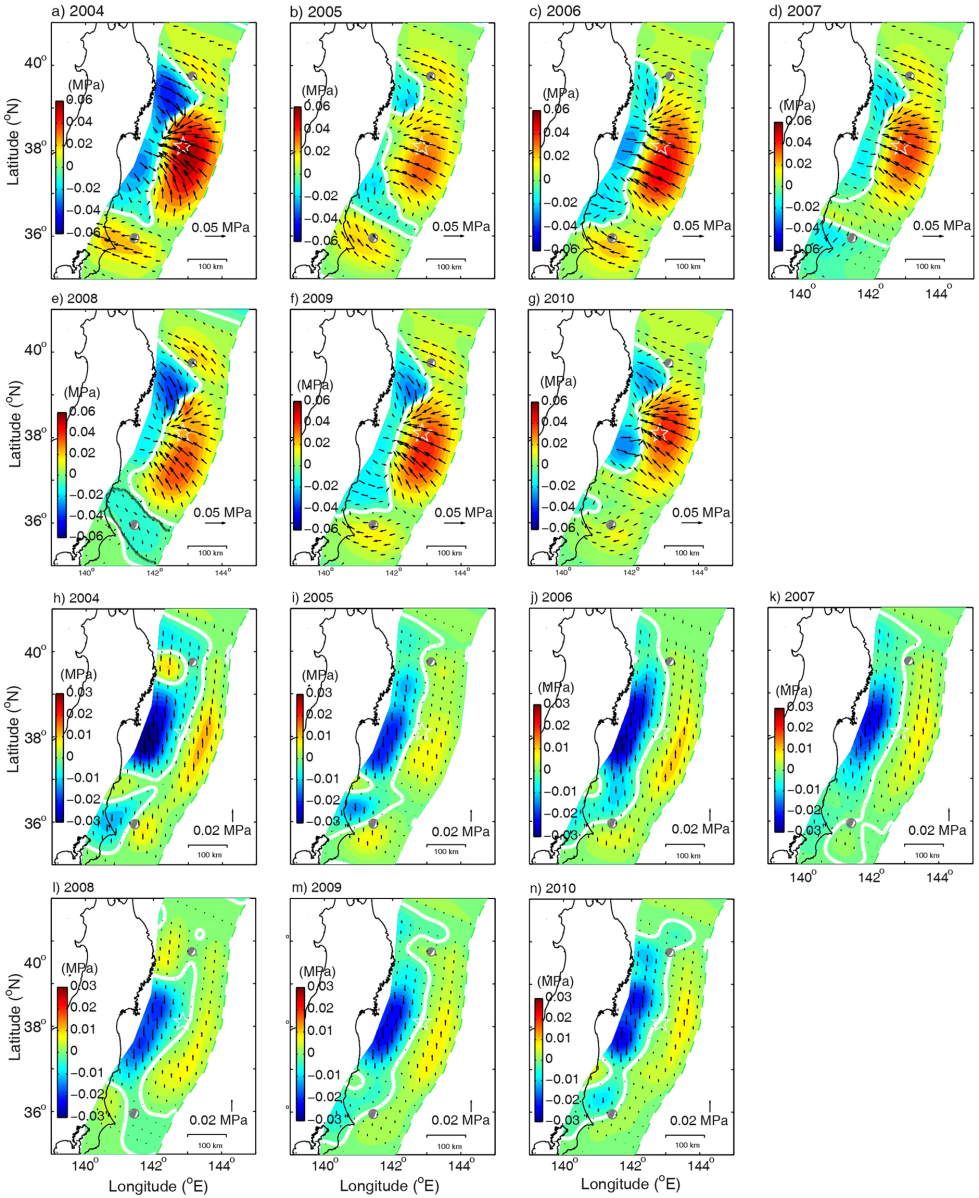
Inverted accumulations of shear stress (**a**–**g**) and normal stress (**h**–**n**) per year from January 1, 2004 to December 31, 2010, respectively.

In order to examine if any significant temporal variations in stress accumulation have occurred during these seven years, we plot in Fig. 3 the stress change for each year. The stress accumulation area of the main shock from year to year is consistent with that of the 7 years. Although the magnitude of the shear and normal stresses varies over time and space, the location of the one-year seismogenic areas is basically the same as that of the accumulated stresses over the seven–years (Fig. 2), which implies that these areas are stable.

**Figure 4:**
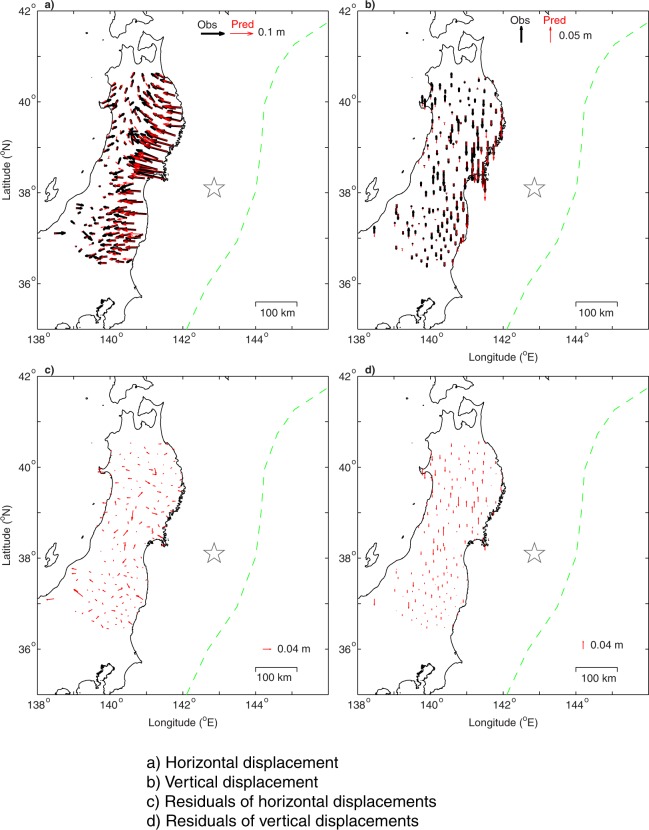
Observed and model predicted cumulative displacements of GNSS site over the period of January 1, 2004 to December 31, 2010, as a result of interseismic stress accumulation prior to the Tohoku-Oki earthquake. (**a**) Horizontal displacement vectors. (**b**) Vertical displacement vectors. (**c**) Residuals between the observed and predicted horizontal displacements. (**d**) Residuals between the observed and predicted vertical displacements.

The surface displacements predicted by the accumulated stress fit the GNSS data observed at 163 stations. The residuals between the observed and the predicted displacements over the seven years are shown in Fig. 4. Both the deviations of the predicted horizontal and vertical displacements are less than those of the accumulated observation errors (See Supplementary Materials), suggesting that the inversion results are reliable. Testing runs (Supplementary material, Figs S3–S6) also show that the inversion results are robust.

## Discussions

### Normal stress and the seismogenic asperity

According to the Coulomb failure criterion^17^, failure occurs on a fault when the shear stress exceeds the strength of the fault. For faults characterized by a given frictional coefficient, the strength of the fault is proportional to the normal stress. Thus the greater the normal stress on the fault the higher the fault strength and the greater amount of strain energy that may be accumulated on the fault. Figure 2 shows that the areas of greater accumulations of shear and normal stresses match the main rupture areas of the M9.0 Tohoku-Oki earthquake and the two largest (Mw 7.4 and 7.8) aftershocks. Based on these results we define the seismogenic asperity as the area of greater accumulations of shear and normal stresses, which is different but consistent with previous definition of ‘asperity’ as the area on which large post-seismic slip occurs^18^.

### Reliability of the identified seismogenic asperity and the stress release zone

The identification of the seismogenic asperity of the Tohoku-Oki earthquake, with the fault area of greater shear and normal stress accumulations (Fig. 2) has not been reported before. In addition, the two smaller seismogenic asperities identified in this study have not been reported in previous studies. To test their reliability, we compare the seismogenic asperity of this study with the rupture areas obtained from fault slip inversion and from seismisity. Based on the earthquake dislocation model, a interseismic coupling area in the Japan subduction zone was predicted by inverted GPS data^5,6^. But the centers of both predicted areas were much deeper (35–40 km) than that of our seismogenic asperity (~15 km). The agreement of the latter with the rupture area of the mainshock^13^ is strong evidence for the reliability of the present method. Furthermore, the agreement between the two smaller seismogenic asperities predicted in this study also compare well with the areas of the two largest aftershocks. In addition, the predicted depth of the seismogenic asperity of the mainshock by the contour of zero-shear stress is consistent with that of the rupture termination identified^13^, marked by black-dish line in Fig. 2a.

The area of shear stress decrease in Fig. 2a implies strain energy release, which corresponds to the slow slip area estimated by the cumulative slip induced by the small repeating earthquakes^19^. It may also explain the occurrence of slip acceleration (Fig. S1a) between the latitudes of 36.5° and 39.5° and depths between 20 to 60 km predicted by *Mavrommatis et al*.^20^ using the onshore GPS data, and explain the very long-term transient event predicted using the GPS data (Fig. S1b)^21^, which occurred in the similar area as that predicted^20^.

Figure 2 shows a large area of reduced shear stress northwest of the Tohoku-Oki earthquake (marked with blue) where high acceleration of fault slip may be expected. However, much of this area sits within a sliding deceleration area predicted by *Mavrommatis et al*. The different predictions of the two models may be partly attributed to the different time periods of the two studies, while our study is from 2004 to 2011, the study of *Mavrommatis et al*. was from 1984 to 2011.

Because only land-based GNSS data were used, our inversion suffers from the same lack of near-trench constraint as in any other models. We also attempted to use annual seafloor displacements in the inversion, which was provided by the Japan Coast Guard, but these displacements are very difficult to use partly because the observations were performed much less frequently and partly because the data contain large errors. Nevertheless, the tests of resolution powers of the used GNSS data onshore to the seimogenic stress model show that main features are recovered in the stress distribution patterns (Fig. S2); thus the seismogenic asperities determined by the stress accumulations are robust and acceptable. The features of the asperities predicted are stable for the wide ranges of the smoothing factor from 0.03 to 0.1 (Figs S3 and 4) and the constraining coefficient from 0.01 to 0.1 (Figs S5 and 6), this means that the asperities are not affected seriously by the values of the smoothing factors and constraining coefficients in these ranges even if they deviate in the knee points, the optimal points on the trade-off curves in Fig. S7.

### Effect of the normal stress change on depth of the seismogenic asperities

The present model also predicts that the normal stress changes with time on the megathrust prior to the Tohoku-Oki earthquake (Fig. 2b). As a result, the predicted frictional strength increases in the up-dip portion from the hypocenter and decrease in the down-dip portion. Thus the change in frictional strength shifts the seismogenic asperity to a shallower depth on the megathrust. This result is consistent with the occurrence of the Tohoku earthquake and with the fact that most large subduction zone earthquakes occur at shallow depths^15,22^. We infer that the tendency for giant earthquakes to include at the shallow portion of megathrusts may be attributed to the increase of the normal stress there, which leads to an increase of fault shear strength and allows more elastic strain energy to accumulate in the shallow portion of the megathrust and to prepare for the next big earthquake. In the previous inversions for slip deficit, the relative normal displacement is usually neglected and thus the change of normal stress on the fault cannot be predicted. This may be one of reasons why these studies predicted the coupling areas at significantly greater depths than the actual rupture depth of the mainshock, as noted earlier.

### Effect of the mainshock on the seismogenic asperities of large aftershocks

The two relatively minor asperities of shear and normal stresses to the south and the north of the main asperity match the hypocenter locations of the two largest aftershocks with *M*_w_ 7.8 and 7.4 that occurred within half an hour after the mainshock. Using forward calculation, we evaluate the changes of the shear and normal stresses imparted by the main shocks to be, respectively, −0.005 and 0.008 MPa on the southern seismogenic asperity, and 0.037 and −0.008 MPa on the northern seismogenic asperity. These changes are more than an order of magnitude smaller than those accumulated in the seismogenic asperities of the two aftershocks in seven years. Thus the stresses imparted by the main shock on these two asperities could not have significantly changed their stress state.

On the one hand, it may be interesting to inquire if the aftershocks were triggered by the main shock. Our evaluation shows that the mainshock causes the shear stress to increase and the normal stress to decrease in the area of the northern asperity of the Mw 7.4 aftershock, consistent with the hypothesis that this aftershock may be triggered by the main shock. On the other hand, the main shock causes the shear stress to decrease and the normal stress to increase in the area of the southern asperity. These changes are inconsistent with the hypothesis that the main shock triggered the Mw 7.8 aftershock. It may also be worth noting that the stress change and the change of the Coulomb failure stress imparted to the two asperities by the mainshock are one order of magnitude smaller than that accumulated in one year. Thus more studies are needed to reveal the triggering mechanism. Processes such as pore pressure changes.

### A mechanical hint for earthquake forecast

Strain energy in Earth’s crust accumulates during loading and is released during faulting. If we assume that the seismogenic asperities of an active fault experiences the same phases of deformation, we may, in principle, infer the current phase of the seismogenic asperity by examining the change of the stress accumulation both in time and space, if detailed GNSS observations are available. Furthermore, because earthquake magnitude is proportional to the area of the rupture zone, study of the size of the seismogenic asperity may also allow an estimate of the magnitude of the potential earthquake. Thus, in theory, the method proposed in this study has the potential for making short-term forecast of earthquakes on active faults if detailed geodetic observations are available for the determination of the time-dependent changes of stresses on the seismogenic asperities.

### Concluding remarks

We find three seismogenic asperities on the megathrust of the 2011 Mw 9.0 Tohoku earthquake by inverting the displacement data at 163 GPS stations of the GEONET from 2004 to 2011, which match the rupture asperities of the mainshock and two largest aftershocks. We also determined the interseismic changes of the normal stress on the fault; it increases in the up-dip portion (shallower depth) from the hypocenter and decreases in the down-dip portion. We infer that the tendency for giant earthquakes to occur at the shallow portion of megathrusts may be attributed to the increase of the normal stress there, which leads to an increase of fault shear strength and allows more elastic strain energy to accumulate to prepare for the next big earthquake. Although the present model cannot predict the timing of damaging earthquakes, it may be useful to predict the locations of the seismogenic asperities of future large earthquakes and may thus be useful for suggesting further investigations that may bear on the timing of the earthquake.

Several important questions remain unanswered. While the accumulations of stresses on the seismogenic asperity of the main shock seem to be steady over the seven years before the earthquake (Fig. 3), the stresses on the asperity of the aftershock south of the main shock changed significantly in 2008 (Fig. 3e). The cause for such changes is currently uncertain. Also uncertain is why the hypocenters of the main shock of the 2011 Tohoku earthquake occurs close to the zero-normal stress contour, why the rupture termination occurs close to the zero-shear stress contour^13^, and what mechanism controls the locations of the zero-shear stress and zero-normal stress and their formation. Further studies are obviously required to answer these questions.

## Methods

*Xie and Cai*^13^ proposed a method to invert for fault stress change based on an earthquake stress model, by which they inverted the land-based coseismic GNSS displacements to determine the coseismic stress drop associated with the 2011 *M*_W_ 9.0 Tohoku-Oki earthquake. The model consists of two elastic tectonic blocks in contact along an interface representing the subduction megathrust that hosts the future earthquake. The use of an interface to represent a fault zone may be justified by the scale of the study, which is many orders of magnitude greater than the fault zone thickness^23^. The normal displacement across the fault is continuous, and the stress vectors on the two sides have the same magnitude but are opposite in direction.

We summarize below the inverse method^13^ to invert for the stress change: (1) set up an earthquake stress model in the FEM, which includes an interface (fault); (2) calculate the numerical Green’s function solutions (displacement fields) due to an unit stress on each sub-fault surface; (3) construct the Green’s function matrix by superposition of the sub-fault solutions at specified observation sites; (4) invert for stress accumulation on the fault surfaces with GNSS observations; and (5) determine the seismogenic asperity. The detailed inversion procedure is given in *Xie and Cai*.

In this work, we use the same method and the pre-seismic GNSS data to invert for fault stress accumulation on the megathrust over a period of seven years prior to the Tohoku-Oki earthquake.

Our study region includes the megathrust fault that hosted the 2011 *M*_w_ 9.0 Tohoku-Oki earthquake and two largest aftershocks (Fig. 1a). In this study, we have not considered the effect of the Japan Triple Junction on the stress change on the megathrust because it is far from the Mw 9.0 Tohoku-Oki earthquake and the earthquake occurred along the Japan Trench well to the north of the junction and did not involve the other two trenches. Considering at least 30 years passed from the large interplate earthquakes (Mw > 7.5) in the Japan Trench in the past century to the Tohoku-Oki earthquake^9^, we neglect the effect of the upper mantle stress relax induced by the earthquakes on the seismogenic area of the Tohoku-Oki earthquake, because the time is much longer than the relax time of the mantle stress, which is about 1 year for taking the viscosity of the upper mantle as 3 × 10^19^ Pa·s^24^, Young’s modulus and Poison’s ratio, 166 GPa and 0.27 in average (Table 1), respectively.

**Table 1:** Material parameters used in the finite element model. *V*_P_ is P wave velocity, *V*_S_, S wave velocity, *E*, Young’s modulus, *ν*, Poisson’s ratio. Elastic parameters in the oceanic crust (depth from 5 to 12 km) and slap (thickness 7 km) are assigned as those in the lower continental crust; in the uppermost mantle, same elastic parameters are assumed beneath the continent and the ocean.

	Depth	*V*_P_	*V*_S_	Density	*E*	*ν*
	(km)	(km/s)	(km/s)	(kg/m^3^)	(GPa)	
Upper Continental crust	10	6	3.55	2600	81	0.2307
Lower Continental crust	25	6.6	3.76	2900	103	0.2597
Uppermost mantle	40	7.6	4.3	3200	150	0.2646
	90	7.7	4.3	3200	151	0.2734
	200	8.3	4.6	3400	184	0.2783

The finite element model of the study region (Fig. 1b) is set up in a Cartesian coordinate system (*x*, *y*, *z*), with the *x-* and *y-*axes being horizontal and normal to and along the Japan Trench, respectively, and the *z-*axis being vertical (Fig. 1a,b). The depth of the Japan Trench in the model is set at a uniform depth of 7 km, although the actual trench depth exhibits some along-strike variations. The model fault has a dip of 11° between 7–47 km deep and 30° at depths greater than 47 km; this geometry is consistent with the centroid moment tensor of the main shock from GCMT (global centroid moment tensor) Catalog and the earthquake hypocenters in the subduction zone^16,25^. The fault surface (Fig. 1) is divided into 170 planar sub-faults (Fig. 1d), on which stress accumulation prior to the Tohoku-Oki earthquake is determined by inverting the GNSS data. The model dimensions along *x*, *y* and *z* directions are 1700 km, 1200 km and 200 km, respectively. The lateral model boundaries are set far from the rupture area of the fault so that their effect on the calculated stress is small. Displacements on the lateral and bottom boundaries are fixed in the normal directions but free in the tangential directions; the top surface is traction free. These choices of boundary conditions may be justified because the stress accumulation from the inversion represent the *changes* that are superimposed on a background of quasi steady-state stress that does not change during the time interval of this study.

The mechanical material parameters^26–29^ used in the model are listed in Table 1.

We use the F3 solution of GNSS data provided by Geospatial Information Authority of Japan. Following *Yoshioka et al*.^30^, we removed steps associated with large earthquakes (Mw ≥ 6.9) and antenna exchange, annual and semi-annual components, and common-mode errors from original time series using a least square method, with fixed stations at Murakami, Kurokawa, and Shibata in Niigata Prefecture. Additionally, we removed the postseismic displacements associated with the 2003 Tokachi-Oki (*M*_w_ 7.8), the 2005 Miyagiken-Oki (*M*_w_ 7.1) and the 2008 Miyagi-Iwate nairiku (*M*_w_ 6.9) earthquakes. We then obtain the best-fit parameters from the corrected time series at each component at each station, using ABIC^31^. Finally, we take the difference between the end and the beginning of each year of the best-fit curve to obtain the yearly displacement fields, which are provided in Supplementary Information: Table S2.

Using these parameters, an optimal smoothing factor, and an optimal constraining coefficient (Supplementary Information: Fig. S7), We invert the three-component displacements at the 163 GNSS sites in the Tohoku district from January 1, 2004 to December 31, 2010 for stress change on the fault over this period.

## Supplementary information

Supplimentary Information
