# Supplementary material for: Fault stress inversion reveals seismogenic asperity of the 2011 Mw 9.0 Tohoku-Oki earthquake
Source: Sci Rep. 2019 Aug 19;9:11987. doi: 10.1038/s41598-019-47992-x (PMC6700084; doi:10.1038/s41598-019-47992-x)
Supplement: Supplementary file 1 — Supplimentary Information [file 41598_2019_47992_MOESM1_ESM.docx]

**SUPPLEMENTARY INFORMATION**

**Title: Fault stress inversion reveals seismogenic asperity of the 2011 Mw9.0 Tohoku-Oki earthquake**

Zhoumin Xie^1,2^, Yongen Cai^3*^, Chi-yuen Wang^4^, Shoichi Yoshioka^5,6^ and Momo Tanaka^6^

^1^Institute of Crustal Dynamics, China Earthquake Administration, Beijing 100085, China.

^2^Department of Earth, Planetary, and Space Sciences, University of California, Los Angeles, CA 90095-156702, USA.

^3^Institute of Theoretical and Applied Geophysics, School of Earth and Space Sciences, Peking University, Beijing 100871, China

^4^Department of Earth and Planetary Science, University of California, Berkeley, CA 94720, United States

^5^Research Center for Urban Safety and Security, Kobe University, Kobe 657-8501, Japan

^6^Department of Planetology, Graduate School of Science, Kobe University, Kobe 657-8501, Japan.

*Corresponding author: Yongen Cai ([yongen@pku.edu.cn)](mailto:yongen@pku.edu.cn))

**SUPPLEMENTARY INFORMATION**

The deviations of the predicted and observed displacements are provided.

Figure S1 shows relationship of the decrease area of the shear stress with the areas of acceleration slip and very long-term transient event.

The resolution power of the data from GNSS for the seismogenic stress model is checked by the shear and normal stress checkerboards in Figure S2.

The effects of smoothing factors and constraining coefficients on the inverted stress accumulation are shown in Figures S3–S6 for the used observation data, material properties, and geometry of the seismogenic stress model of the Tohoku-Oki earthquake. They show the stress accumulation zones predicted are stable for that the ranges of smoothing factors are more than 0.03 and less than 0.1 and that the constraining coefficients are more than 0.01amd less than 0.1, which means that even though the selected factors and coefficients deviate from their optimal values on the trade-off curves, their effects do not change the main features of the stress accumulation zones.

The optimal smoothing factor and the constraining coefficient are searched by using trade-off curves shown in Figure S7.

Tables S1 is the GNSS data used in the study.

Tables S2 is the results of shear and normal stresses predicted by the GNSS data，respectively .

**The deviations between the predicted and observed displacements**

Defining the root mean square (RMS) of the observational error *e*_i_ as $E=\frac{1}{N}\sum_{i=1}^{N} e_{2}^{2}$ were *N* (=163) is the number of observation stations. The RMS of the derivation between the predicted displacements $d_{i}^{prd}$and the observed displacement $d_{i}^{obs}$ defined as$D=\frac{1}{N}\sum_{i=1}^{N} (d_{i}^{prd}-d_{i}^{obs})^{2}$ , respectively, we obtain the horizontal $E_{h}=0.0137$ and the vertical $E_{v}=0.0444$ , respectively, and the horizontal $D_{h}=0.0107$ and $D_{v}=0.0148$.

**Figure S1**


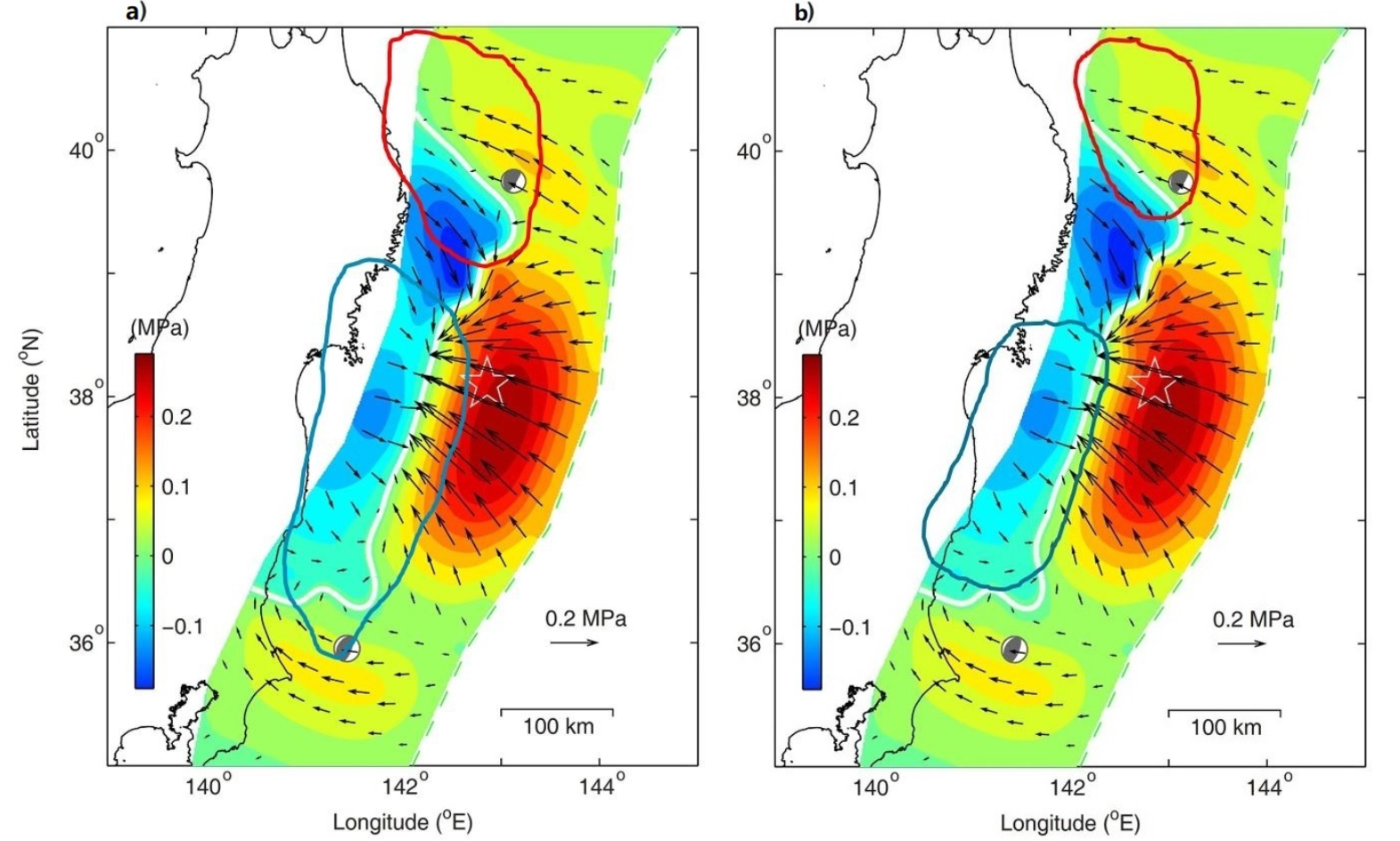


**Figure S1.** Relationship of the decrease area of the shear stress with the areas of acceleration slip and very long-term transient event. a) The areas surrounded by the blue and red lines represent the areas of acceleration slip and deceleration slip^24^, respectively. b) The areas surrounded by the blue and red lines represent the distributions of the forward slip (toward to the trench) by the very long-term transient event and the backslip by the northern source^25^, respectively.

**Figure S2**


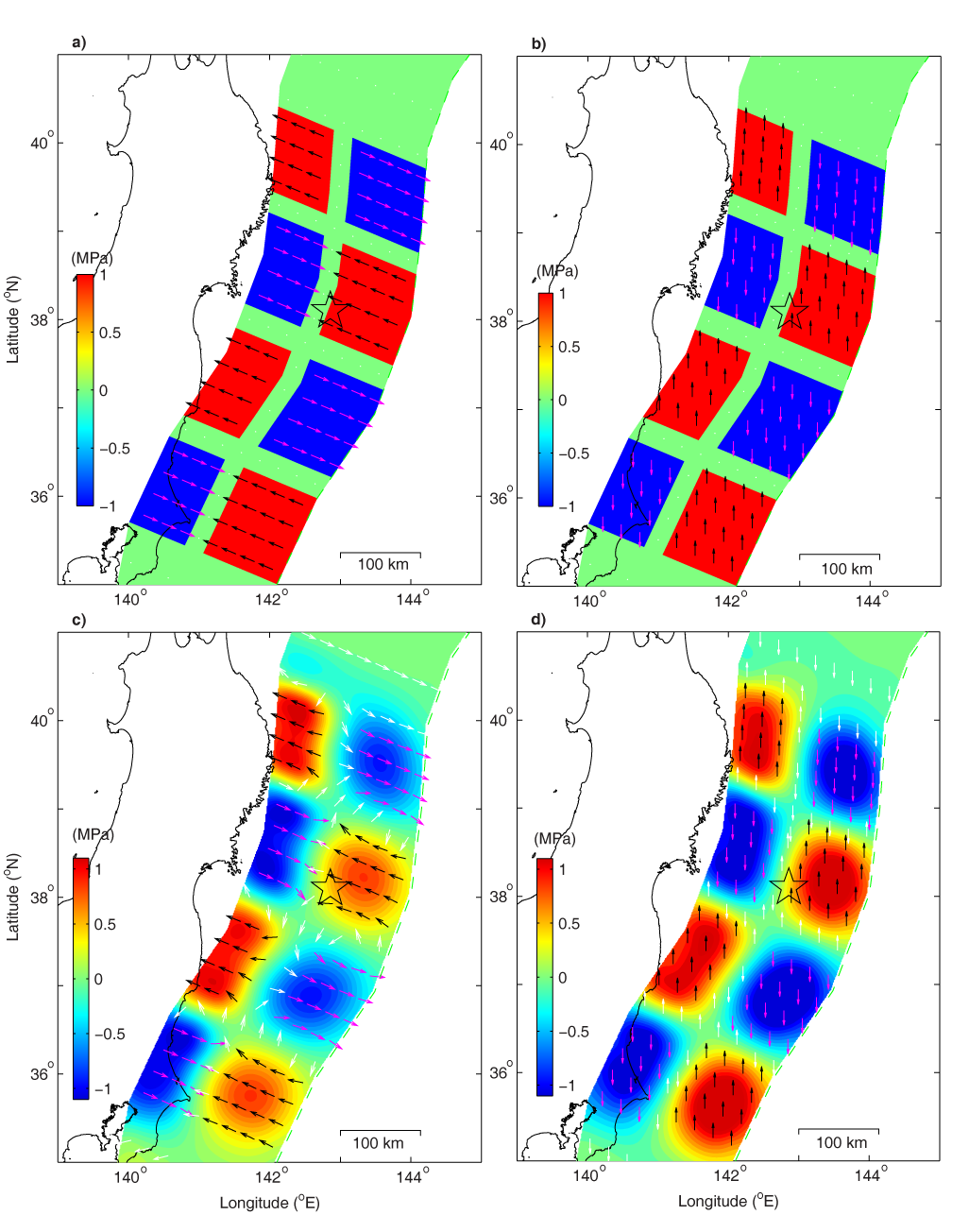


**Figure S2.** Resolution power of stress checkerboard. (a) and (b) Checkerboards with one unit of shear stress and normal stress, respectively. (c) and (d) Recovered shear stress and normal stress, respectively.

**Figure S3**

**
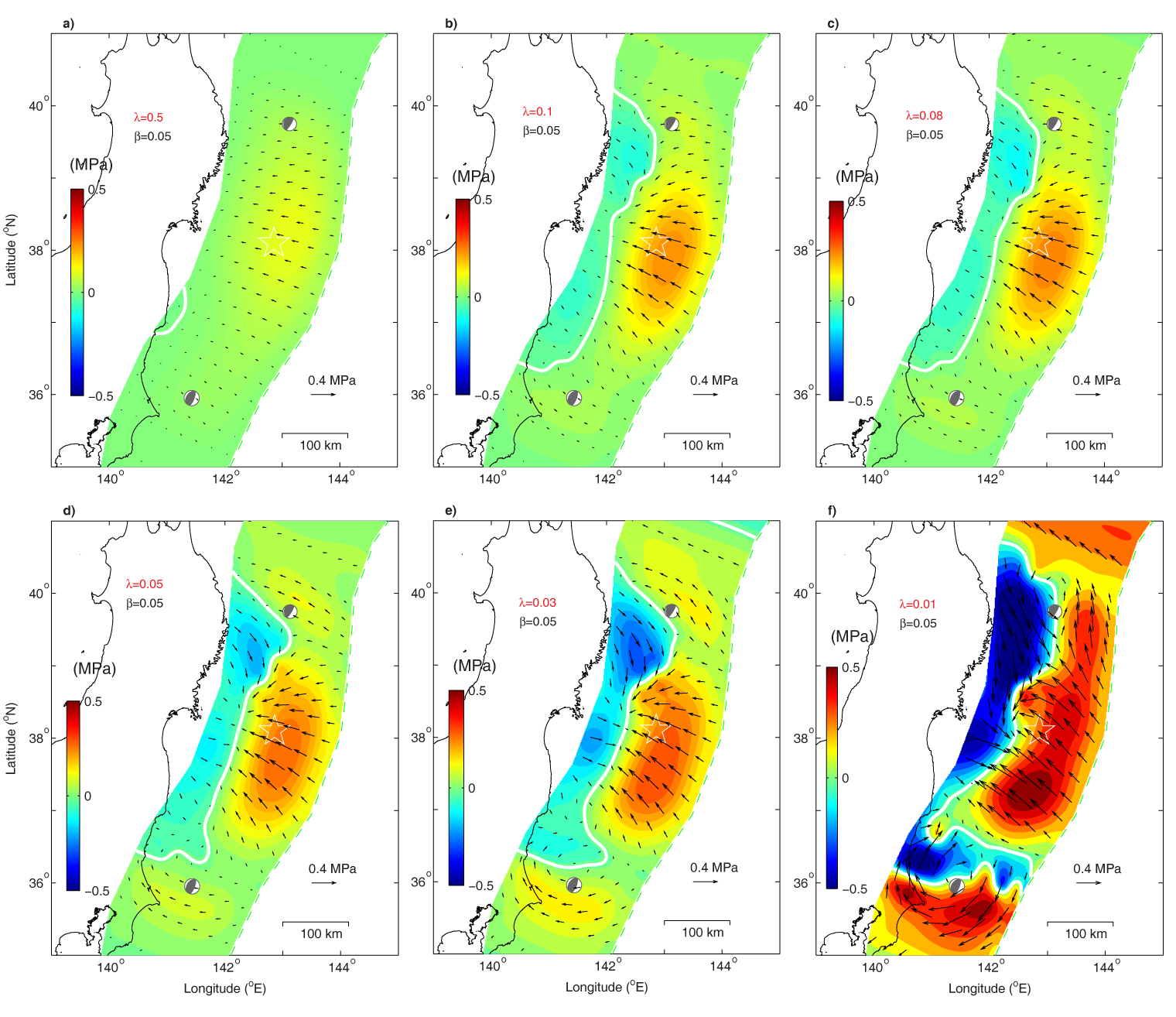
**

**Figure S3.** Sensitivity of the predicted shear stress distribution to the smoothing factor $\lambda$ for the fixed optimal constraining coefficient $\beta=0.05$. **a) - f)** For the inverted shear stresses. The over-smoothed and under-smoothed corresponding to $\lambda>0.05$ and $\lambda<0.05$, respectively. The optimal model **d)** is found from the trade-off curves (Figure S7).

**Figure S4**

**
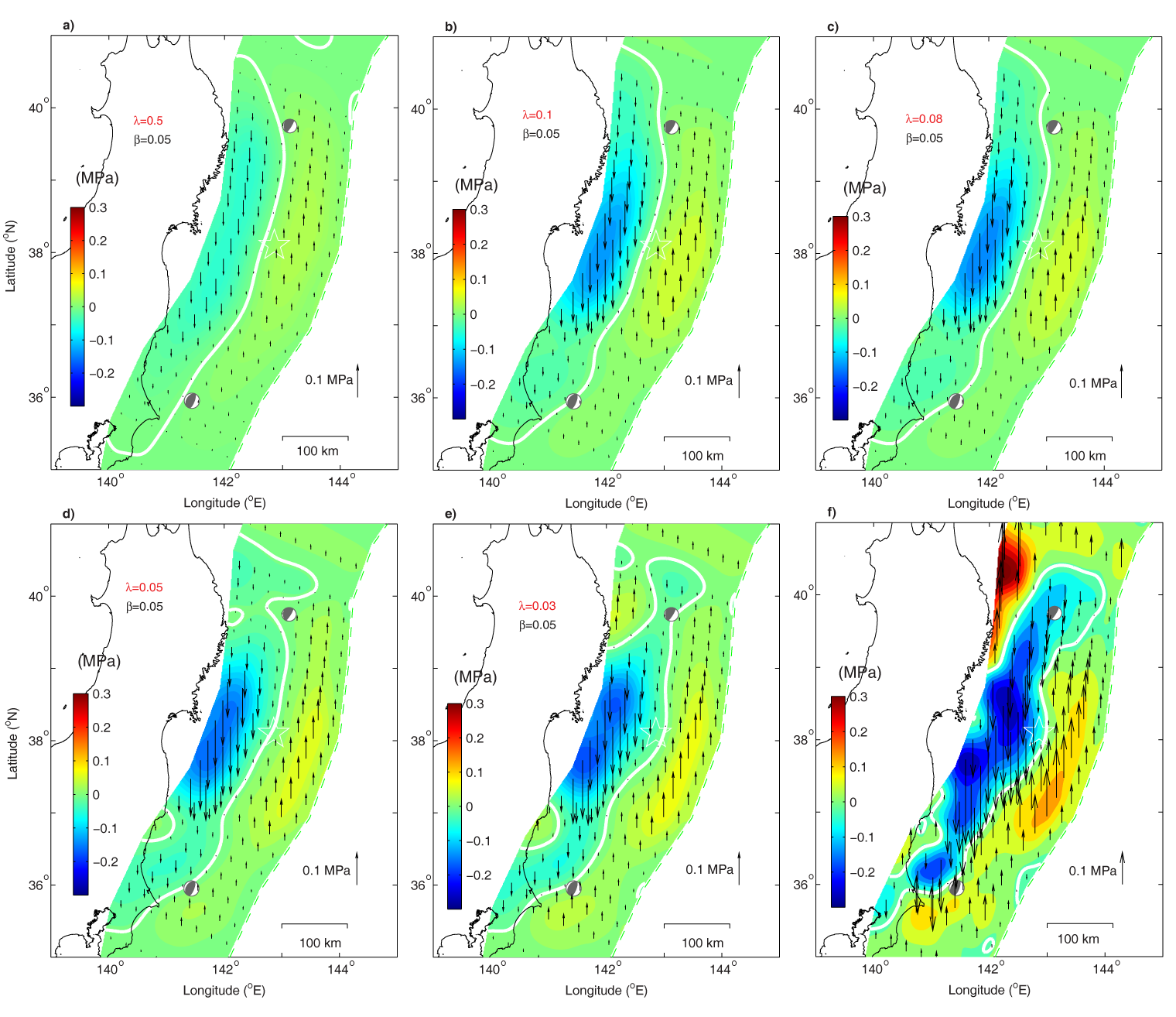
**

**Figure S4**. Sensitivity of the predicted normal stress distribution to the smoothing factor $\lambda$ for the fixed optimal constraining factor $\beta=0.05$. **a) - f)** the inverted normal stresses. The over-smoothed and under-smoothed corresponding to $\lambda>0.05$ and $\lambda<0.05$, respectively. The Optimal model **d)** is found from the trade-off curves (Figure S7).

**Figure S5**

**
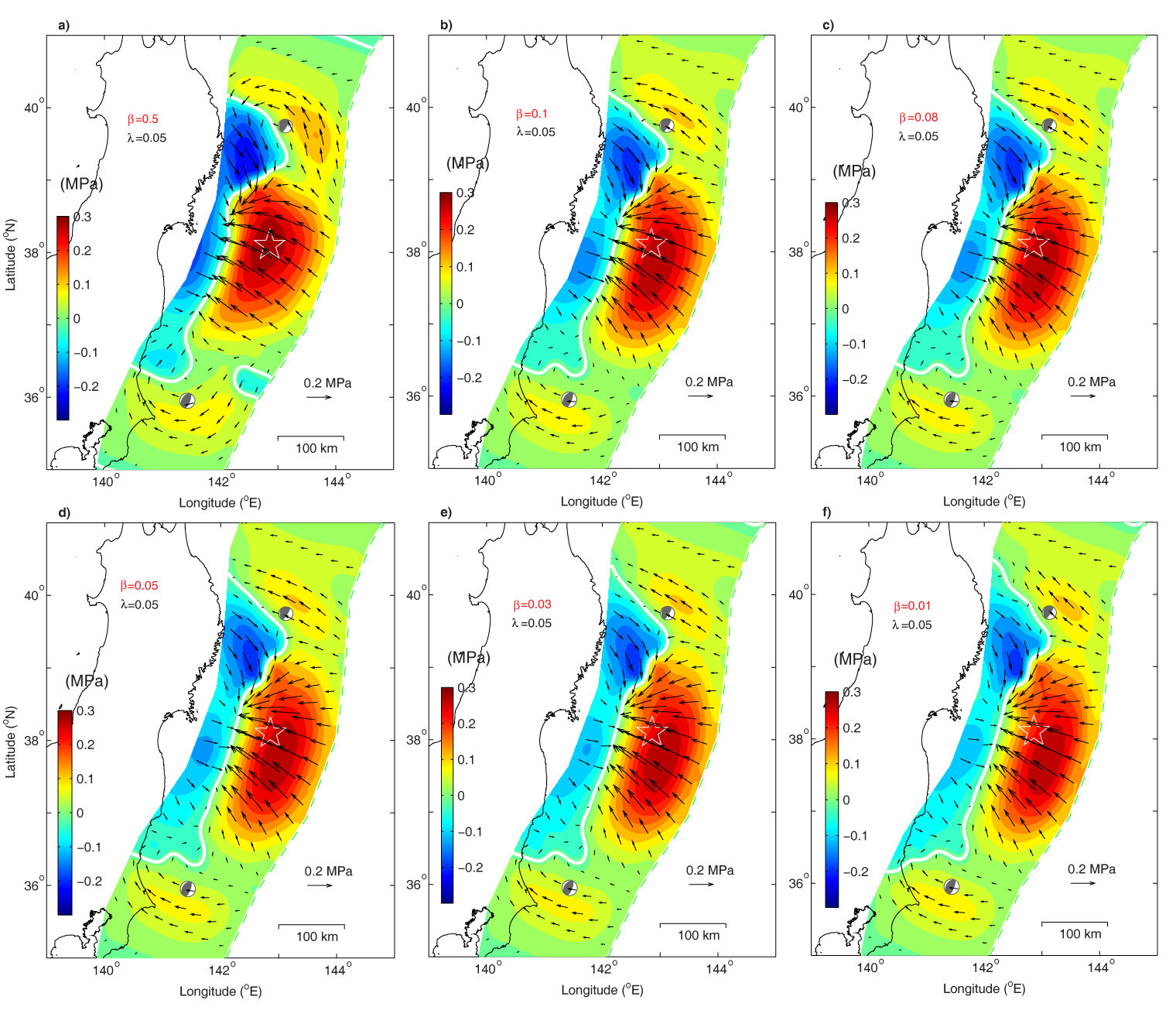
**

**Figure S5.** Sensitivity of the predicted shear stress distribution to the constraining coefficient $\beta$ for the fixed optimal smoothing factor $\lambda=0.05$. a) - f) For the inverted shear stresses. The optimal model d) is found from the trade-off curves (Figure S7).

**Figure S6**


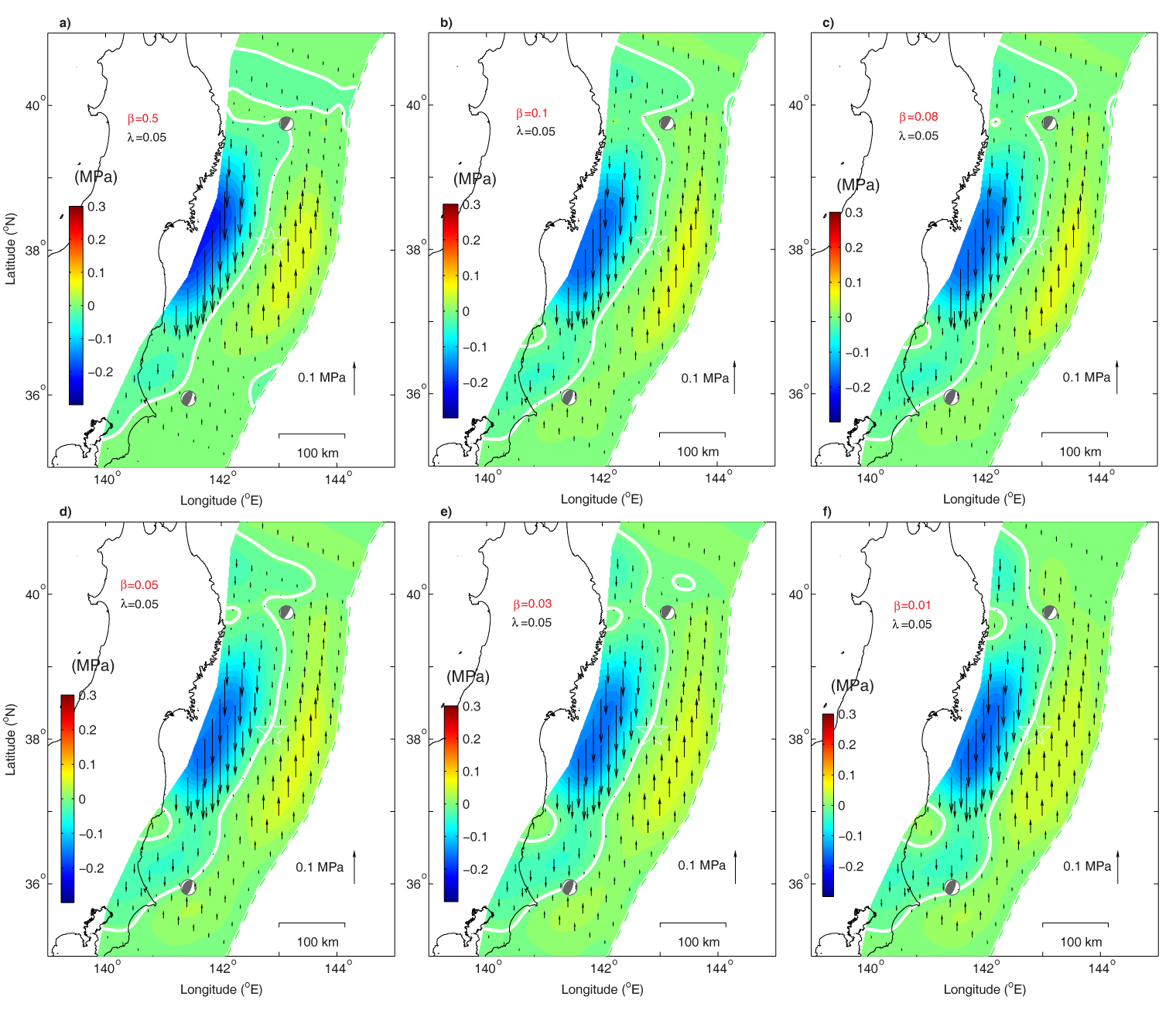


**Figure S6**. Sensitivity of the predicted stress distribution to the constraining coefficient $\beta$ for the fixed optimal smoothing factor $\lambda=0.05$. a) - f) For the inverted normal stresses. The optimal model d) is found from the trade-off curves (Figure S7).

**Figure S7**


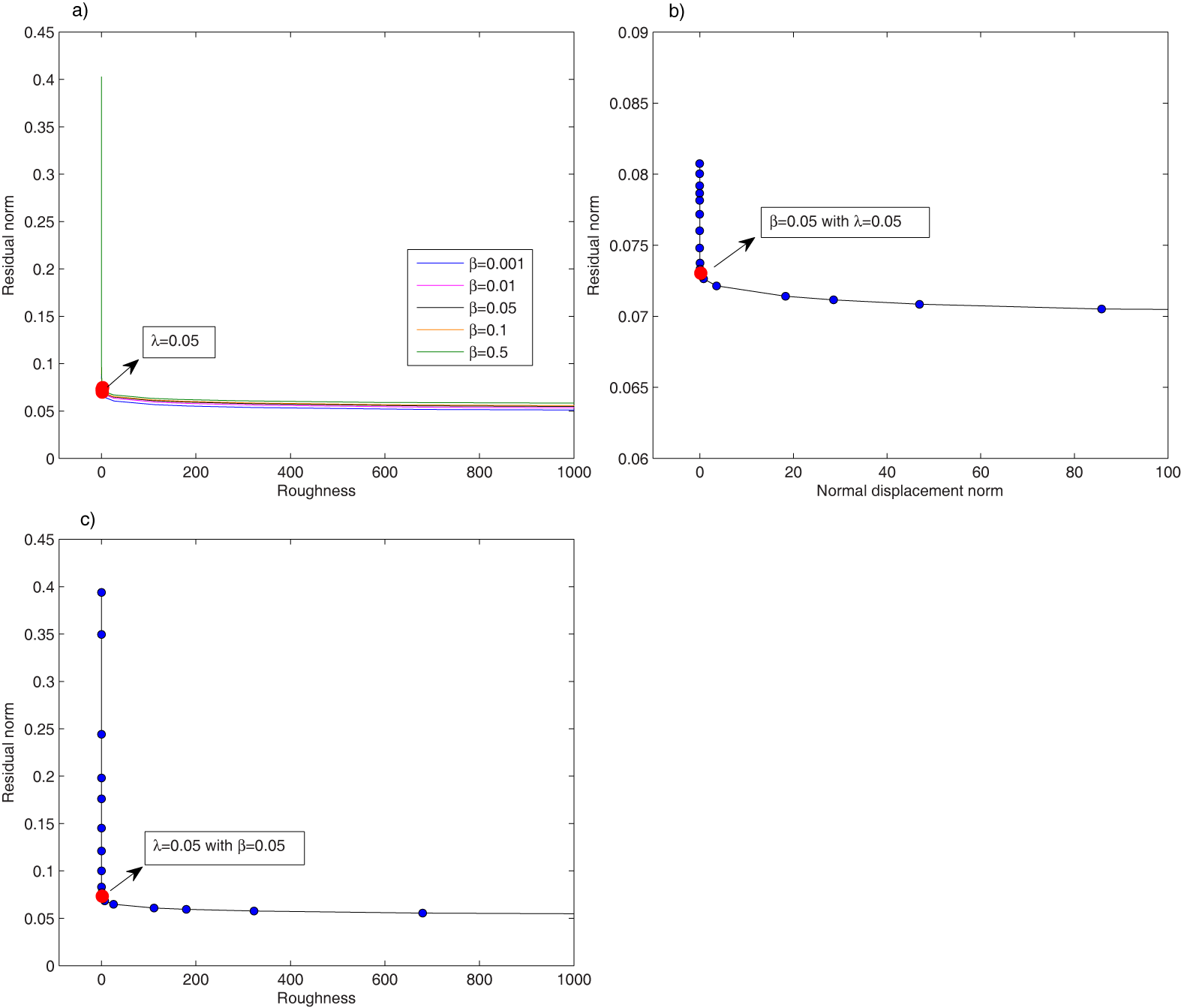


**Figure S7**. Trade-off curves of the smoothing factor $\lambda$ and the constraining coefficient $\beta$. **a)** Trade-off curves with the varying $\lambda$ from 0.001 to 10 for each $\beta$ varying from 0.001 to 0.5. The determined optimal $\lambda$ is 0.05. The horizontal axis for smoothing degree, $\sum_{j=1}^{3M} \left| \left( \mathbf{LT} \right)_{j} \right|^{2}$, the vertical axis for fitting degree, $\sum_{i=1}^{3N} \left| \left( \mathbf{d}-\mathbf{GT} \right)_{i} \right|^{2}$, where **L** is a Laplacian differential operator, **T**, the stress change to be solved on the fault, **d**, the displacement vector on the observation stations, *M* and *N*, the number of sub-faults and observation stations, respectively. **b)** The trade-off curve with varying $\beta$ from 0.001 to 10.0 for the optimal $\lambda=0.05$. The optimal $\beta$ is 0.05. The horizontal axis is the squared normal displacement，*w*，on the ruptured fault plane, $\sum_{j=1}^{M} \left| w_{j} \right|^{2}$, and the vertical axis is the same as a). **c)** The trade-off curve picked up from a) with different $\lambda$ for the fixed optimal $\beta=0.05$.

**Table S1**

Table S1. Displacement change observed by GNSS onshore from January 1, 2004 to December 31, 2010

| Station | Latitude | Longitude | Displace(m) | Displace(m) | Displace(m) |  |  |  |
| --- | --- | --- | --- | --- | --- | --- | --- | --- |
|  |  |  | Eastward | Northward | Upward | Error_east | Error_north | Error_up |
| 1 | 36.45658 | 139.90251 | -0.034374 | -0.000118 | -0.011324 | 0.008589 | 0.009813 | 0.035289 |
| 2 | 40.44763 | 140.94159 | -0.00848 | 0.032943 | -0.003333 | 0.015306 | 0.017113 | 0.057669 |
| 3 | 36.47585 | 140.38569 | -0.033715 | 0.003562 | -0.011215 | 0.009742 | 0.011154 | 0.038758 |
| 4 | 36.53763 | 139.75279 | -0.030626 | 0.010282 | -0.007489 | 0.009504 | 0.010799 | 0.039416 |
| 5 | 36.53993 | 140.61184 | -0.023353 | -0.013224 | -0.008524 | 0.012281 | 0.014242 | 0.040105 |
| 6 | 36.54222 | 140.17931 | -0.026503 | -0.000332 | -0.001544 | 0.009578 | 0.011218 | 0.036315 |
| 7 | 36.59895 | 139.92347 | -0.030774 | -0.002379 | -0.008863 | 0.009354 | 0.010788 | 0.037027 |
| 8 | 36.62417 | 139.49059 | -0.025343 | -0.00228 | -0.010294 | 0.012655 | 0.014238 | 0.044135 |
| 9 | 36.65057 | 140.29342 | -0.040163 | 0.011729 | -0.015766 | 0.013826 | 0.016093 | 0.04024 |
| 10 | 36.66598 | 139.61925 | -0.028815 | -0.017961 | -0.007029 | 0.011509 | 0.013101 | 0.041799 |
| 11 | 39.01211 | 141.40085 | -0.087101 | 0.028561 | -0.013455 | 0.010727 | 0.012146 | 0.044075 |
| 12 | 36.69246 | 140.15769 | -0.037658 | 0.002057 | -0.011679 | 0.009103 | 0.0104 | 0.035459 |
| 13 | 38.74322 | 141.31786 | -0.092961 | 0.039835 | -0.060364 | 0.009971 | 0.011263 | 0.038891 |
| 14 | 36.74303 | 140.4976 | -0.041024 | 0.002301 | -0.006963 | 0.010256 | 0.011477 | 0.03689 |
| 15 | 36.76969 | 139.2249 | -0.011756 | -0.012076 | 0.000713 | 0.014191 | 0.015885 | 0.044017 |
| 16 | 36.77637 | 139.85426 | -0.024806 | -0.010249 | -0.007572 | 0.010427 | 0.011897 | 0.039692 |
| 17 | 36.77896 | 140.29649 | -0.043218 | 0.00138 | -0.007007 | 0.009531 | 0.010907 | 0.036747 |
| 18 | 36.80031 | 140.75391 | -0.048863 | -0.000833 | -0.001792 | 0.011022 | 0.012325 | 0.038652 |
| 19 | 36.83736 | 139.06085 | -0.000011 | -0.002722 | -0.009475 | 0.01802 | 0.019942 | 0.057722 |
| 20 | 36.85424 | 140.03926 | -0.045946 | 0.000024 | -0.010303 | 0.010866 | 0.012266 | 0.039549 |
| 21 | 36.86209 | 140.41327 | -0.047334 | -0.00275 | -0.007824 | 0.009951 | 0.011212 | 0.037494 |
| 22 | 36.87225 | 139.50531 | 0.004017 | 0.00475 | 0.001234 | 0.014573 | 0.016332 | 0.050919 |
| 23 | 36.93262 | 140.69015 | -0.049231 | 0.002991 | -0.010323 | 0.011222 | 0.012592 | 0.040496 |
| 24 | 36.95231 | 140.16476 | -0.038663 | 0.00006 | 0.000782 | 0.01075 | 0.012205 | 0.041979 |
| 25 | 36.97958 | 139.80575 | -0.017612 | -0.012645 | 0.0028 | 0.013472 | 0.01528 | 0.043339 |
| 26 | 37.01845 | 140.84165 | -0.063523 | -0.002192 | -0.016754 | 0.010963 | 0.01233 | 0.039929 |
| 27 | 37.02358 | 140.37627 | -0.031706 | 0.000045 | -0.0103 | 0.012231 | 0.013583 | 0.040625 |
| 28 | 37.03694 | 139.40223 | -0.015848 | -0.004765 | 0.025829 | 0.023942 | 0.026712 | 0.071983 |
| 29 | 37.0893 | 140.56164 | -0.047145 | -0.00686 | -0.019901 | 0.010284 | 0.01166 | 0.036657 |
| 30 | 37.09071 | 140.90251 | -0.037821 | -0.01738 | -0.011134 | 0.011534 | 0.013067 | 0.037508 |
| 31 | 37.09725 | 139.58616 | -0.02177 | 0.003081 | 0.008808 | 0.012641 | 0.014242 | 0.048108 |
| 32 | 37.1247 | 140.03541 | -0.035797 | -0.014833 | -0.018883 | 0.024515 | 0.026682 | 0.063136 |
| 33 | 37.92059 | 139.87941 | -0.006765 | -0.004826 | 0.015035 | 0.011924 | 0.013549 | 0.04426 |
| 34 | 37.12629 | 140.25993 | -0.047162 | -0.012307 | -0.011955 | 0.012476 | 0.01405 | 0.044704 |
| 35 | 37.18309 | 140.716 | -0.059829 | 0.003527 | -0.016256 | 0.010089 | 0.011401 | 0.039511 |
| 36 | 37.21056 | 140.45097 | -0.050589 | 0.00103 | -0.010069 | 0.010496 | 0.01184 | 0.03944 |
| 37 | 37.26107 | 139.87143 | -0.007005 | -0.013876 | 0.008609 | 0.01166 | 0.013149 | 0.041637 |
| 38 | 37.28149 | 139.50377 | -0.012392 | -0.001151 | -0.000446 | 0.010534 | 0.012049 | 0.036989 |
| 39 | 37.29304 | 140.21275 | -0.05358 | -0.015057 | -0.022702 | 0.013992 | 0.015579 | 0.044752 |
| 40 | 37.3028 | 139.36435 | -0.009301 | -0.002838 | 0.007599 | 0.013577 | 0.014967 | 0.041016 |
| 41 | 37.31987 | 139.02025 | -0.013839 | 0.011521 | 0.003084 | 0.015663 | 0.016978 | 0.038521 |
| 42 | 37.32547 | 140.66211 | -0.05364 | 0.001649 | -0.018066 | 0.012878 | 0.014293 | 0.041854 |
| 43 | 37.32621 | 139.68635 | -0.017524 | 0.00091 | 0.001294 | 0.012292 | 0.013843 | 0.048907 |
| 44 | 37.36182 | 140.32439 | -0.035215 | 0.000412 | -0.01704 | 0.01702 | 0.018583 | 0.048176 |
| 45 | 37.42451 | 140.13588 | -0.034713 | -0.004579 | -0.001149 | 0.012558 | 0.013933 | 0.042363 |
| 46 | 37.44487 | 140.46403 | -0.054294 | 0.015423 | -0.017104 | 0.012991 | 0.014453 | 0.048654 |
| 47 | 37.46129 | 139.83552 | -0.023309 | -0.001683 | 0.002169 | 0.012268 | 0.013875 | 0.042098 |
| 48 | 37.47346 | 139.52863 | 0.008104 | -0.016671 | 0.017421 | 0.010979 | 0.012414 | 0.038455 |
| 49 | 37.53369 | 141.00661 | -0.073845 | 0.004093 | -0.039139 | 0.011389 | 0.012852 | 0.044095 |
| 50 | 37.54034 | 139.11145 | 0.009961 | 0.005824 | -0.016682 | 0.00992 | 0.011383 | 0.042827 |
| 51 | 37.56055 | 140.75508 | -0.054176 | -0.006895 | -0.027726 | 0.011591 | 0.01337 | 0.043511 |
| 52 | 37.10709 | 138.45736 | 0.047518 | 0.001886 | 0.021464 | 0.010515 | 0.01221 | 0.04544 |
| 53 | 37.15762 | 139.24711 | 0.03951 | -0.038894 | -0.008341 | 0.016473 | 0.018599 | 0.068956 |
| 54 | 37.56695 | 140.07268 | -0.026221 | -0.007309 | 0.008766 | 0.011349 | 0.01258 | 0.042794 |
| 55 | 37.59109 | 140.57165 | -0.053408 | -0.001435 | -0.023739 | 0.013158 | 0.01486 | 0.047392 |
| 56 | 37.61595 | 140.20565 | -0.039094 | -0.017467 | 0.004038 | 0.012717 | 0.014549 | 0.052465 |
| 57 | 37.62073 | 140.3729 | -0.050349 | -0.002968 | -0.018714 | 0.033554 | 0.035585 | 0.068751 |
| 58 | 37.6437 | 139.79151 | -0.009825 | -0.00384 | 0.003142 | 0.011517 | 0.013003 | 0.041466 |
| 59 | 37.6623 | 139.05912 | 0.024877 | -0.000528 | -0.018224 | 0.007853 | 0.009091 | 0.031526 |
| 60 | 37.66929 | 139.97266 | -0.02163 | 0.004458 | -0.016071 | 0.010926 | 0.012272 | 0.043144 |
| 61 | 37.68331 | 140.46649 | -0.04846 | -0.006256 | -0.009258 | 0.01724 | 0.019035 | 0.056184 |
| 62 | 37.68591 | 139.47789 | 0.005083 | -0.007337 | 0.002707 | 0.009344 | 0.010678 | 0.037618 |
| 63 | 37.75206 | 139.07391 | 0.0286 | -0.005392 | -0.010226 | 0.007434 | 0.008516 | 0.030291 |
| 64 | 37.80067 | 140.90808 | -0.067287 | -0.001864 | -0.029166 | 0.01162 | 0.013372 | 0.041809 |
| 65 | 37.8246 | 140.72766 | -0.062297 | 0.001738 | -0.027544 | 0.01371 | 0.015347 | 0.046919 |
| 66 | 37.8335 | 140.44679 | -0.030945 | -0.00346 | 0.010492 | 0.015025 | 0.016645 | 0.054469 |
| 67 | 37.96361 | 140.09353 | -0.020366 | 0.003037 | 0.010875 | 0.01122 | 0.012591 | 0.039855 |
| 68 | 37.98441 | 140.64564 | -0.057024 | 0.003972 | -0.015873 | 0.014512 | 0.016151 | 0.048562 |
| 69 | 37.98993 | 140.44257 | -0.019426 | 0.018664 | 0.001726 | 0.014959 | 0.016779 | 0.044718 |
| 70 | 38.02966 | 140.84399 | -0.067671 | 0.00524 | -0.02702 | 0.011641 | 0.013102 | 0.043173 |
| 71 | 38.14618 | 139.74225 | -0.005637 | -0.000605 | 0.01166 | 0.014245 | 0.015685 | 0.042003 |
| 72 | 38.1481 | 140.27114 | -0.026988 | 0.001112 | -0.012749 | 0.012347 | 0.013737 | 0.045465 |
| 73 | 38.17044 | 140.39388 | -0.034692 | -0.03112 | -0.018747 | 0.012899 | 0.014766 | 0.061782 |
| 74 | 38.19754 | 140.07757 | -0.011526 | 0.000586 | 0.025836 | 0.019748 | 0.021445 | 0.054296 |
| 75 | 38.20574 | 140.64285 | -0.05336 | -0.017863 | -0.007454 | 0.020326 | 0.022284 | 0.056427 |
| 76 | 38.28898 | 140.19992 | -0.020984 | -0.001139 | 0.022847 | 0.011951 | 0.01337 | 0.04345 |
| 77 | 38.30119 | 141.50069 | -0.13164 | 0.030961 | -0.039863 | 0.010661 | 0.012113 | 0.038485 |
| 78 | 38.31749 | 140.95418 | -0.071001 | 0.006923 | -0.031894 | 0.01128 | 0.012688 | 0.042271 |
| 79 | 38.33103 | 140.36609 | -0.036924 | 0.003496 | 0.011308 | 0.011172 | 0.012718 | 0.041869 |
| 80 | 38.42506 | 141.21291 | -0.086113 | 0.012966 | -0.022254 | 0.00958 | 0.010976 | 0.039794 |
| 81 | 38.43434 | 140.09463 | -0.015717 | -0.000326 | 0.019895 | 0.014817 | 0.016475 | 0.046041 |
| 82 | 38.4492 | 141.44116 | -0.10405 | 0.012382 | -0.037496 | 0.011688 | 0.013364 | 0.039415 |
| 83 | 38.49677 | 140.36515 | -0.042984 | -0.018022 | -0.047181 | 0.011879 | 0.013571 | 0.044952 |
| 84 | 38.50984 | 141.3044 | -0.122656 | 0.035099 | -0.039609 | 0.00991 | 0.011285 | 0.037972 |
| 85 | 38.53948 | 141.14754 | -0.08803 | 0.010575 | -0.037263 | 0.010363 | 0.011752 | 0.037888 |
| 86 | 38.57181 | 140.72772 | -0.04013 | 0.010138 | -0.011901 | 0.011933 | 0.013287 | 0.041116 |
| 87 | 38.59404 | 139.83177 | 0.010671 | 0.000694 | 0.002085 | 0.018614 | 0.020071 | 0.059051 |
| 88 | 38.62841 | 140.22051 | 0.009497 | 0.026462 | 0.007567 | 0.015554 | 0.017619 | 0.057249 |
| 89 | 38.66063 | 141.16072 | -0.07451 | 0.04133 | -0.026432 | 0.009537 | 0.010773 | 0.039116 |
| 90 | 38.6827 | 141.44937 | -0.12301 | 0.025418 | -0.038501 | 0.011207 | 0.012627 | 0.039448 |
| 91 | 38.68614 | 141.00434 | -0.073286 | 0.02014 | -0.028571 | 0.010279 | 0.01177 | 0.04168 |
| 92 | 38.70117 | 139.67453 | 0.015638 | 0.003766 | -0.001 | 0.008351 | 0.009775 | 0.037255 |
| 93 | 38.74894 | 140.80164 | -0.051787 | 0.007499 | 0.005656 | 0.012297 | 0.01379 | 0.04375 |
| 94 | 38.75216 | 140.49726 | -0.019033 | 0.003201 | -0.012212 | 0.01652 | 0.018002 | 0.052197 |
| 95 | 38.75972 | 139.95737 | 0.000247 | -0.001185 | 0.007977 | 0.008597 | 0.009926 | 0.035831 |
| 96 | 38.8946 | 139.80885 | 0.024531 | 0.011471 | -0.002421 | 0.008334 | 0.009481 | 0.033909 |
| 97 | 38.9381 | 140.18347 | 0.003278 | 0.012659 | -0.013204 | 0.015305 | 0.016728 | 0.045526 |
| 98 | 38.99546 | 141.14853 | -0.078805 | 0.034401 | -0.047605 | 0.011977 | 0.013507 | 0.047597 |
| 99 | 39.01597 | 139.92749 | 0.02591 | 0.01486 | 0.004559 | 0.009024 | 0.010311 | 0.036837 |
| 100 | 39.02378 | 141.73985 | -0.094768 | 0.02589 | -0.026485 | 0.01236 | 0.013805 | 0.040551 |
| 101 | 39.0519 | 140.62958 | -0.011767 | 0.068101 | 0.003734 | 0.012273 | 0.014083 | 0.041764 |
| 102 | 39.05438 | 140.44733 | -0.007804 | 0.00931 | -0.013078 | 0.01152 | 0.013253 | 0.044661 |
| 103 | 39.11062 | 141.20392 | -0.056384 | 0.037035 | -0.046463 | 0.013442 | 0.015408 | 0.049262 |
| 104 | 39.12701 | 140.98846 | -0.047563 | 0.033208 | -0.04141 | 0.018983 | 0.02067 | 0.055358 |
| 105 | 39.1462 | 140.71505 | -0.017475 | 0.03217 | -0.004927 | 0.015612 | 0.017309 | 0.054391 |
| 106 | 39.16421 | 140.16198 | 0.008024 | -0.006503 | 0.011313 | 0.018302 | 0.020408 | 0.058998 |
| 107 | 39.1991 | 140.50669 | 0.000797 | 0.020493 | 0.00457 | 0.010075 | 0.011527 | 0.038157 |
| 108 | 39.20606 | 139.90772 | 0.032509 | 0.00852 | -0.001002 | 0.008055 | 0.009258 | 0.034352 |
| 109 | 39.23538 | 141.31167 | -0.053757 | 0.020956 | -0.041488 | 0.010899 | 0.012352 | 0.042368 |
| 110 | 39.25352 | 141.79804 | -0.070259 | 0.023748 | -0.02458 | 0.017023 | 0.019065 | 0.054979 |
| 111 | 39.29675 | 140.27893 | 0.014012 | 0.011541 | 0.004918 | 0.011151 | 0.01253 | 0.04229 |
| 112 | 39.327 | 140.55978 | 0.008762 | 0.017866 | -0.033425 | 0.010456 | 0.01181 | 0.036147 |
| 113 | 39.33806 | 141.53416 | -0.06539 | 0.022297 | 0.004648 | 0.012696 | 0.014233 | 0.041494 |
| 114 | 39.3513 | 140.76918 | -0.037353 | -0.001323 | 0.036708 | 0.01648 | 0.018098 | 0.050378 |
| 115 | 39.3987 | 140.04824 | 0.019011 | 0.009725 | 0.002724 | 0.009045 | 0.010426 | 0.036976 |
| 116 | 39.46469 | 141.293 | -0.055834 | 0.0299 | -0.030451 | 0.011825 | 0.013457 | 0.051059 |
| 117 | 39.50814 | 140.59609 | -0.005345 | 0.01862 | -0.024051 | 0.009594 | 0.011052 | 0.041639 |
| 118 | 39.52757 | 140.05393 | 0.016009 | 0.010432 | 0.010759 | 0.008893 | 0.010207 | 0.034909 |
| 119 | 39.54929 | 140.38662 | 0.003407 | 0.017163 | -0.001046 | 0.008599 | 0.009894 | 0.034272 |
| 120 | 39.55446 | 140.83632 | -0.011033 | 0.021813 | -0.004047 | 0.023487 | 0.025774 | 0.061879 |
| 121 | 39.57239 | 141.93996 | -0.060125 | 0.014825 | -0.010602 | 0.014037 | 0.01574 | 0.049949 |
| 122 | 39.59613 | 141.67529 | -0.058631 | 0.020744 | -0.016098 | 0.014494 | 0.016301 | 0.049308 |
| 123 | 39.59657 | 141.17211 | -0.040758 | 0.027464 | -0.023403 | 0.012668 | 0.014082 | 0.0457 |
| 124 | 39.63937 | 141.42613 | -0.03622 | 0.020633 | -0.028853 | 0.012239 | 0.014311 | 0.045969 |
| 125 | 39.65831 | 140.23421 | 0.022572 | 0.015678 | 0.000098 | 0.009035 | 0.010368 | 0.034301 |
| 126 | 39.70109 | 140.9645 | -0.041195 | 0.036668 | -0.018988 | 0.016444 | 0.018124 | 0.057314 |
| 127 | 39.70268 | 140.73285 | -0.011588 | 0.02334 | -0.015465 | 0.012962 | 0.014626 | 0.04595 |
| 128 | 39.73896 | 141.97022 | -0.05959 | 0.012482 | -0.011273 | 0.012325 | 0.013976 | 0.04458 |
| 129 | 39.74916 | 140.59704 | 0.013395 | 0.018073 | 0.003116 | 0.014059 | 0.015522 | 0.045348 |
| 130 | 39.82581 | 140.04472 | 0.020779 | 0.016209 | 0.020429 | 0.008475 | 0.009689 | 0.034542 |
| 131 | 39.84902 | 141.45259 | -0.039621 | 0.01807 | -0.018686 | 0.01142 | 0.013109 | 0.041925 |
| 132 | 39.84918 | 141.80385 | -0.055655 | 0.010321 | -0.013382 | 0.012881 | 0.014542 | 0.04354 |
| 133 | 39.85125 | 141.16486 | -0.0432 | 0.026622 | -0.018615 | 0.015972 | 0.017878 | 0.053631 |
| 134 | 39.86937 | 141.95054 | -0.064281 | 0.006213 | -0.009766 | 0.011458 | 0.013011 | 0.039778 |
| 135 | 39.89113 | 139.84893 | 0.050174 | 0.011141 | 0.0232 | 0.012796 | 0.014154 | 0.041011 |
| 136 | 39.92139 | 140.53586 | 0.015747 | 0.013704 | 0.004865 | 0.011326 | 0.012867 | 0.046462 |
| 137 | 39.93606 | 140.13238 | 0.011247 | 0.01879 | 0.021122 | 0.008804 | 0.010411 | 0.035044 |
| 138 | 39.95308 | 141.06633 | -0.038594 | 0.026859 | -0.004195 | 0.017522 | 0.019588 | 0.053362 |
| 139 | 39.9679 | 139.77615 | 0.037016 | 0.015402 | 0.023452 | 0.009751 | 0.011191 | 0.036887 |
| 140 | 39.97096 | 141.66218 | -0.051446 | 0.012498 | -0.01108 | 0.013693 | 0.01563 | 0.04834 |
| 141 | 39.98058 | 141.22507 | -0.032753 | 0.051833 | 0.010529 | 0.011915 | 0.014074 | 0.044437 |
| 142 | 40.00688 | 140.40199 | 0.015513 | 0.015301 | 0.018956 | 0.010055 | 0.011527 | 0.038117 |
| 143 | 40.04498 | 140.82291 | 0.000103 | 0.017433 | -0.005443 | 0.019935 | 0.021825 | 0.048579 |
| 144 | 40.0486 | 141.46195 | -0.036121 | 0.020069 | -0.019891 | 0.012088 | 0.013679 | 0.041002 |
| 145 | 40.0997 | 140.0055 | 0.021345 | 0.015253 | 0.007719 | 0.007967 | 0.009174 | 0.033473 |
| 146 | 40.10675 | 141.04646 | -0.019391 | 0.021921 | -0.007301 | 0.012806 | 0.014379 | 0.044264 |
| 147 | 40.13345 | 141.7891 | -0.047295 | 0.013187 | 0.008726 | 0.01191 | 0.013732 | 0.046102 |
| 148 | 40.18332 | 140.40815 | 0.008336 | 0.022424 | 0.01029 | 0.009921 | 0.011689 | 0.048226 |
| 149 | 40.21544 | 140.78733 | -0.002155 | 0.023469 | 0.011737 | 0.010277 | 0.011646 | 0.040071 |
| 150 | 40.2473 | 140.04868 | 0.01729 | 0.014018 | 0.018737 | 0.008147 | 0.009485 | 0.033814 |
| 151 | 40.27104 | 141.48486 | -0.047198 | 0.011063 | -0.00884 | 0.013958 | 0.01549 | 0.044583 |
| 152 | 40.27116 | 140.26366 | 0.007577 | 0.015355 | 0.017177 | 0.008866 | 0.010279 | 0.036326 |
| 153 | 40.29108 | 141.07648 | -0.002005 | 0.014513 | -0.008329 | 0.013284 | 0.014903 | 0.043446 |
| 154 | 40.29115 | 141.29369 | -0.025893 | 0.022167 | -0.002356 | 0.011893 | 0.013475 | 0.040743 |
| 155 | 39.45816 | 141.95526 | -0.064844 | 0.004259 | -0.041251 | 0.013085 | 0.014613 | 0.042389 |
| 156 | 40.32505 | 140.57735 | 0.007923 | 0.029287 | 0.009653 | 0.009977 | 0.011291 | 0.036918 |
| 157 | 40.3394 | 140.02757 | 0.017857 | 0.011172 | 0.010531 | 0.008328 | 0.009538 | 0.034963 |
| 158 | 40.4052 | 141.71314 | -0.051117 | 0.015128 | 0.007837 | 0.012104 | 0.013624 | 0.040665 |
| 159 | 38.90286 | 141.57259 | -0.097308 | 0.026125 | -0.043101 | 0.011536 | 0.012979 | 0.040557 |
| 160 | 40.50876 | 141.33199 | -0.029902 | 0.020639 | -0.007307 | 0.010974 | 0.012497 | 0.041137 |
| 161 | 40.51536 | 141.51128 | -0.038703 | 0.02137 | -0.002726 | 0.010085 | 0.01143 | 0.037546 |
| 162 | 40.52365 | 140.57815 | 0.013239 | 0.022284 | 0.014729 | 0.010435 | 0.011924 | 0.039028 |
| 163 | 39.14309 | 141.57549 | -0.085907 | 0.025705 | -0.029445 | 0.010603 | 0.012027 | 0.042173 |

**Table S2**

**Table S2**. Inverted stresses on the fault surface of the overriding plate. T*_r_*, T*_s_* and T*_n_* (positive for compression) denote the stress components of an inverted stress vector (traction) defined in the local coordinate system *r*-*s*-*n* on the megathrust fault (Figure 1). The direction cosine of *r* axis for all sub-faults is *e_r_*=(−0.9806, 0, −0.1961) defined in the global coordinate system *x*, *y*, *z* (see Figure 1), namely, N67^o^W and dip angle 11^o^. The direction cosine of *s* axis, ***e_s_***, is calculated from $\boldsymbol{e}_{\boldsymbol{n}}\boldsymbol{\times}\boldsymbol{e}_{\boldsymbol{r}}$ **
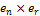
**, where ***e_n_*** (*n_x_*, *n_y_*, *n_z_*) is the direction cosine of *n* axis, vertically pointed to the sub-fault surface. For the stress vectors in the sub-faults from B1 to B19 are expressed in global coordinate system as T*_x_*, T*_y_* and T*_z_* . Error denotes the difference between the inverted stress component obtained by the observation data with and without overlying noise.

| Subf. | | Lon | | Lat | | Depth | | Stress vector | | | | Direction cosine of Tn | | | |
| --- | --- | --- | --- | --- | --- | --- | --- | --- | --- | --- | --- | --- | --- | --- | --- |
|  |  | |  | |  | | T*r* | | T*_s_* | T*n* | *n*_x_ | | *n*_y_ | *n*_z_ |  |
| # | (°) | | (°) | | (km) | | （MPa） | | （MPa） | （MPa） |  | |  |  |  |
| 1 | 142.04 | | 35.17 | | 9.5 | | 0.0257 | | 0.0116 | 0.0069 | -0.1961 | | 0.0065 | 0.9806 |  |
| 2 | 142.19 | | 35.42 | | 9.5 | | 0.0351 | | 0.0193 | 0.0094 | -0.1961 | | 0.0065 | 0.9806 |  |
| 3 | 142.33 | | 35.66 | | 9.5 | | 0.0290 | | 0.0178 | 0.0078 | -0.1961 | | 0.0065 | 0.9806 |  |
| 4 | 142.47 | | 35.90 | | 9.5 | | 0.0190 | | 0.0052 | 0.0058 | -0.1961 | | 0.0065 | 0.9806 |  |
| 5 | 142.65 | | 36.14 | | 9.5 | | 0.0187 | | -0.0154 | 0.0066 | -0.1960 | | 0.0382 | 0.9799 |  |
| 6 | 142.85 | | 36.38 | | 9.5 | | 0.0347 | | -0.0367 | 0.0119 | -0.1960 | | 0.0382 | 0.9799 |  |
| 7 | 143.05 | | 36.62 | | 9.5 | | 0.0633 | | -0.0507 | 0.0200 | -0.1960 | | 0.0382 | 0.9799 |  |
| 8 | 143.25 | | 36.85 | | 9.5 | | 0.0950 | | -0.0538 | 0.0285 | -0.1960 | | 0.0382 | 0.9799 |  |
| 9 | 143.42 | | 37.11 | | 9.5 | | 0.1210 | | -0.0475 | 0.0318 | -0.1961 | | -0.0089 | 0.9805 |  |
| 10 | 143.55 | | 37.39 | | 9.5 | | 0.1357 | | -0.0319 | 0.0353 | -0.1961 | | -0.0089 | 0.9805 |  |
| 11 | 143.68 | | 37.66 | | 9.5 | | 0.1386 | | -0.0119 | 0.0363 | -0.1961 | | -0.0089 | 0.9805 |  |
| 12 | 143.81 | | 37.94 | | 9.5 | | 0.1302 | | 0.0099 | 0.0346 | -0.1961 | | -0.0089 | 0.9805 |  |
| 13 | 143.89 | | 38.20 | | 9.5 | | 0.1111 | | 0.0266 | 0.0318 | -0.1958 | | -0.0568 | 0.9790 |  |
| 14 | 143.93 | | 38.44 | | 9.5 | | 0.0872 | | 0.0355 | 0.0258 | -0.1958 | | -0.0568 | 0.9790 |  |
| 15 | 143.97 | | 38.68 | | 9.5 | | 0.0648 | | 0.0321 | 0.0202 | -0.1958 | | -0.0568 | 0.9790 |  |
| 16 | 144.00 | | 38.92 | | 9.5 | | 0.0507 | | 0.0181 | 0.0157 | -0.1958 | | -0.0568 | 0.9790 |  |
| 17 | 144.03 | | 39.16 | | 9.5 | | 0.0480 | | -0.0007 | 0.0134 | -0.1957 | | -0.0667 | 0.9784 |  |
| 18 | 144.05 | | 39.40 | | 9.5 | | 0.0527 | | -0.0149 | 0.0129 | -0.1957 | | -0.0667 | 0.9784 |  |
| 19 | 144.07 | | 39.63 | | 9.5 | | 0.0528 | | -0.0182 | 0.0127 | -0.1957 | | -0.0667 | 0.9784 |  |
| 20 | 144.10 | | 39.87 | | 9.5 | | 0.0360 | | -0.0114 | 0.0078 | -0.1957 | | -0.0667 | 0.9784 |  |
| 21 | 141.78 | | 35.26 | | 14.5 | | 0.0464 | | 0.0169 | 0.0103 | -0.1961 | | 0.0065 | 0.9806 |  |
| 22 | 141.93 | | 35.50 | | 14.5 | | 0.0631 | | 0.0279 | 0.0163 | -0.1961 | | 0.0065 | 0.9806 |  |
| 23 | 142.07 | | 35.75 | | 14.5 | | 0.0509 | | 0.0258 | 0.0171 | -0.1961 | | 0.0065 | 0.9806 |  |
| 24 | 142.21 | | 35.99 | | 14.5 | | 0.0308 | | 0.0069 | 0.0160 | -0.1961 | | 0.0065 | 0.9806 |  |
| 25 | 142.38 | | 36.23 | | 14.5 | | 0.0279 | | -0.0245 | 0.0170 | -0.1960 | | 0.0382 | 0.9799 |  |
| 26 | 142.59 | | 36.47 | | 14.5 | | 0.0538 | | -0.0581 | 0.0244 | -0.1960 | | 0.0382 | 0.9799 |  |
| 27 | 142.79 | | 36.70 | | 14.5 | | 0.1016 | | -0.0812 | 0.0384 | -0.1960 | | 0.0382 | 0.9799 |  |
| 28 | 142.99 | | 36.94 | | 14.5 | | 0.1542 | | -0.0872 | 0.0550 | -0.1960 | | 0.0382 | 0.9799 |  |
| 29 | 143.16 | | 37.20 | | 14.5 | | 0.1971 | | -0.0785 | 0.0632 | -0.1961 | | -0.0089 | 0.9805 |  |
| 30 | 143.29 | | 37.47 | | 14.5 | | 0.2229 | | -0.0533 | 0.0667 | -0.1961 | | -0.0089 | 0.9805 |  |
| 31 | 143.42 | | 37.75 | | 14.5 | | 0.2281 | | -0.0193 | 0.0682 | -0.1961 | | -0.0089 | 0.9805 |  |
| 32 | 143.55 | | 38.03 | | 14.5 | | 0.2128 | | 0.0191 | 0.0681 | -0.1961 | | -0.0089 | 0.9805 |  |
| 33 | 143.63 | | 38.28 | | 14.5 | | 0.1807 | | 0.0494 | 0.0616 | -0.1958 | | -0.0568 | 0.9790 |  |
| 34 | 143.67 | | 38.53 | | 14.5 | | 0.1390 | | 0.0663 | 0.0493 | -0.1958 | | -0.0568 | 0.9790 |  |
| 35 | 143.70 | | 38.77 | | 14.5 | | 0.0992 | | 0.0606 | 0.0385 | -0.1958 | | -0.0568 | 0.9790 |  |
| 36 | 143.74 | | 39.01 | | 14.5 | | 0.0751 | | 0.0356 | 0.0311 | -0.1958 | | -0.0568 | 0.9790 |  |
| 37 | 143.77 | | 39.25 | | 14.5 | | 0.0728 | | 0.0027 | 0.0288 | -0.1957 | | -0.0667 | 0.9784 |  |
| 38 | 143.79 | | 39.49 | | 14.5 | | 0.0833 | | -0.0213 | 0.0301 | -0.1957 | | -0.0667 | 0.9784 |  |
| 39 | 143.81 | | 39.72 | | 14.5 | | 0.0859 | | -0.0255 | 0.0257 | -0.1957 | | -0.0667 | 0.9784 |  |
| 40 | 143.83 | | 39.96 | | 14.5 | | 0.0600 | | -0.0144 | 0.0106 | -0.1957 | | -0.0667 | 0.9784 |  |
| 41 | 141.52 | | 35.35 | | 19.5 | | 0.0613 | | 0.0154 | 0.0171 | -0.1961 | | 0.0065 | 0.9806 |  |
| 42 | 141.66 | | 35.59 | | 19.5 | | 0.0818 | | 0.0263 | 0.0250 | -0.1961 | | 0.0065 | 0.9806 |  |
| 43 | 141.81 | | 35.83 | | 19.5 | | 0.0616 | | 0.0249 | 0.0239 | -0.1961 | | 0.0065 | 0.9806 |  |
| 44 | 141.95 | | 36.08 | | 19.5 | | 0.0308 | | 0.0058 | 0.0189 | -0.1961 | | 0.0065 | 0.9806 |  |
| 45 | 142.12 | | 36.32 | | 19.5 | | 0.0247 | | -0.0274 | 0.0162 | -0.1960 | | 0.0382 | 0.9799 |  |
| 46 | 142.32 | | 36.55 | | 19.5 | | 0.0589 | | -0.0644 | 0.0198 | -0.1960 | | 0.0382 | 0.9799 |  |
| 47 | 142.53 | | 36.79 | | 19.5 | | 0.1223 | | -0.0908 | 0.0299 | -0.1960 | | 0.0382 | 0.9799 |  |
| 48 | 142.73 | | 37.03 | | 19.5 | | 0.1908 | | -0.0985 | 0.0415 | -0.1960 | | 0.0382 | 0.9799 |  |
| 49 | 142.90 | | 37.29 | | 19.5 | | 0.2454 | | -0.0887 | 0.0440 | -0.1961 | | -0.0089 | 0.9805 |  |
| 50 | 143.03 | | 37.56 | | 19.5 | | 0.2782 | | -0.0599 | 0.0440 | -0.1961 | | -0.0089 | 0.9805 |  |
| 51 | 143.16 | | 37.84 | | 19.5 | | 0.2850 | | -0.0181 | 0.0436 | -0.1961 | | -0.0089 | 0.9805 |  |
| 52 | 143.29 | | 38.11 | | 19.5 | | 0.2648 | | 0.0317 | 0.0440 | -0.1961 | | -0.0089 | 0.9805 |  |
| 53 | 143.37 | | 38.37 | | 19.5 | | 0.2204 | | 0.0746 | 0.0430 | -0.1958 | | -0.0568 | 0.9790 |  |
| 54 | 143.41 | | 38.61 | | 19.5 | | 0.1612 | | 0.0981 | 0.0359 | -0.1958 | | -0.0568 | 0.9790 |  |
| 55 | 143.44 | | 38.85 | | 19.5 | | 0.1044 | | 0.0909 | 0.0289 | -0.1958 | | -0.0568 | 0.9790 |  |
| 56 | 143.48 | | 39.10 | | 19.5 | | 0.0713 | | 0.0568 | 0.0251 | -0.1958 | | -0.0568 | 0.9790 |  |
| 57 | 143.51 | | 39.34 | | 19.5 | | 0.0719 | | 0.0134 | 0.0261 | -0.1957 | | -0.0667 | 0.9784 |  |
| 58 | 143.53 | | 39.57 | | 19.5 | | 0.0938 | | -0.0164 | 0.0264 | -0.1957 | | -0.0667 | 0.9784 |  |
| 59 | 143.55 | | 39.81 | | 19.5 | | 0.1065 | | -0.0212 | 0.0170 | -0.1957 | | -0.0667 | 0.9784 |  |
| 60 | 143.57 | | 40.05 | | 19.5 | | 0.0781 | | -0.0104 | 0.0012 | -0.1957 | | -0.0667 | 0.9784 |  |
| 61 | 141.26 | | 35.44 | | 24.5 | | 0.0682 | | 0.0077 | 0.0226 | -0.1961 | | 0.0065 | 0.9806 |  |
| 62 | 141.40 | | 35.68 | | 24.5 | | 0.0894 | | 0.0162 | 0.0273 | -0.1961 | | 0.0065 | 0.9806 |  |
| 63 | 141.54 | | 35.92 | | 24.5 | | 0.0616 | | 0.0167 | 0.0166 | -0.1961 | | 0.0065 | 0.9806 |  |
| 64 | 141.69 | | 36.16 | | 24.5 | | 0.0205 | | 0.0015 | 0.0038 | -0.1961 | | 0.0065 | 0.9806 |  |
| 65 | 141.86 | | 36.41 | | 24.5 | | 0.0085 | | -0.0270 | 0.0002 | -0.1960 | | 0.0382 | 0.9799 |  |
| 66 | 142.06 | | 36.64 | | 24.5 | | 0.0435 | | -0.0588 | 0.0065 | -0.1960 | | 0.0382 | 0.9799 |  |
| 67 | 142.27 | | 36.88 | | 24.5 | | 0.1117 | | -0.0819 | 0.0180 | -0.1960 | | 0.0382 | 0.9799 |  |
| 68 | 142.47 | | 37.12 | | 24.5 | | 0.1839 | | -0.0894 | 0.0268 | -0.1960 | | 0.0382 | 0.9799 |  |
| 69 | 142.63 | | 37.37 | | 24.5 | | 0.2401 | | -0.0815 | 0.0249 | -0.1961 | | -0.0089 | 0.9805 |  |
| 70 | 142.76 | | 37.65 | | 24.5 | | 0.2741 | | -0.0550 | 0.0215 | -0.1961 | | -0.0089 | 0.9805 |  |
| 71 | 142.89 | | 37.93 | | 24.5 | | 0.2836 | | -0.0110 | 0.0170 | -0.1961 | | -0.0089 | 0.9805 |  |
| 72 | 143.02 | | 38.20 | | 24.5 | | 0.2641 | | 0.0461 | 0.0141 | -0.1961 | | -0.0089 | 0.9805 |  |
| 73 | 143.11 | | 38.46 | | 24.5 | | 0.2130 | | 0.0995 | 0.0158 | -0.1958 | | -0.0568 | 0.9790 |  |
| 74 | 143.14 | | 38.70 | | 24.5 | | 0.1409 | | 0.1292 | 0.0131 | -0.1958 | | -0.0568 | 0.9790 |  |
| 75 | 143.18 | | 38.94 | | 24.5 | | 0.0710 | | 0.1208 | 0.0096 | -0.1958 | | -0.0568 | 0.9790 |  |
| 76 | 143.22 | | 39.18 | | 24.5 | | 0.0336 | | 0.0791 | 0.0088 | -0.1958 | | -0.0568 | 0.9790 |  |
| 77 | 143.25 | | 39.42 | | 24.5 | | 0.0434 | | 0.0278 | 0.0124 | -0.1957 | | -0.0667 | 0.9784 |  |
| 78 | 143.27 | | 39.66 | | 24.5 | | 0.0844 | | -0.0059 | 0.0131 | -0.1957 | | -0.0667 | 0.9784 |  |
| 79 | 143.29 | | 39.90 | | 24.5 | | 0.1147 | | -0.0118 | 0.0044 | -0.1957 | | -0.0667 | 0.9784 |  |
| 80 | 143.31 | | 40.14 | | 24.5 | | 0.0899 | | -0.0036 | -0.0063 | -0.1957 | | -0.0667 | 0.9784 |  |
| 81 | 141.00 | | 35.52 | | 29.5 | | 0.0680 | | -0.0049 | 0.0188 | -0.1961 | | 0.0065 | 0.9806 |  |
| 82 | 141.14 | | 35.77 | | 29.5 | | 0.0888 | | -0.0020 | 0.0154 | -0.1961 | | 0.0065 | 0.9806 |  |
| 83 | 141.28 | | 36.01 | | 29.5 | | 0.0571 | | 0.0001 | -0.0055 | -0.1961 | | 0.0065 | 0.9806 |  |
| 84 | 141.43 | | 36.25 | | 29.5 | | 0.0086 | | -0.0081 | -0.0221 | -0.1961 | | 0.0065 | 0.9806 |  |
| 85 | 141.60 | | 36.49 | | 29.5 | | -0.0124 | | -0.0240 | -0.0213 | -0.1960 | | 0.0382 | 0.9799 |  |
| 86 | 141.80 | | 36.73 | | 29.5 | | 0.0108 | | -0.0410 | -0.0109 | -0.1960 | | 0.0382 | 0.9799 |  |
| 87 | 142.00 | | 36.97 | | 29.5 | | 0.0643 | | -0.0549 | 0.0005 | -0.1960 | | 0.0382 | 0.9799 |  |
| 88 | 142.21 | | 37.21 | | 29.5 | | 0.1191 | | -0.0622 | 0.0046 | -0.1960 | | 0.0382 | 0.9799 |  |
| 89 | 142.37 | | 37.46 | | 29.5 | | 0.1600 | | -0.0616 | -0.0008 | -0.1961 | | -0.0089 | 0.9805 |  |
| 90 | 142.50 | | 37.74 | | 29.5 | | 0.1876 | | -0.0449 | -0.0060 | -0.1961 | | -0.0089 | 0.9805 |  |
| 91 | 142.63 | | 38.01 | | 29.5 | | 0.2021 | | -0.0045 | -0.0130 | -0.1961 | | -0.0089 | 0.9805 |  |
| 92 | 142.76 | | 38.29 | | 29.5 | | 0.1929 | | 0.0554 | -0.0191 | -0.1961 | | -0.0089 | 0.9805 |  |
| 93 | 142.85 | | 38.55 | | 29.5 | | 0.1478 | | 0.1165 | -0.0165 | -0.1958 | | -0.0568 | 0.9790 |  |
| 94 | 142.88 | | 38.79 | | 29.5 | | 0.0743 | | 0.1506 | -0.0152 | -0.1958 | | -0.0568 | 0.9790 |  |
| 95 | 142.92 | | 39.03 | | 29.5 | | 0.0013 | | 0.1411 | -0.0144 | -0.1958 | | -0.0568 | 0.9790 |  |
| 96 | 142.95 | | 39.27 | | 29.5 | | -0.0327 | | 0.0939 | -0.0103 | -0.1958 | | -0.0568 | 0.9790 |  |
| 97 | 142.98 | | 39.51 | | 29.5 | | -0.0078 | | 0.0381 | -0.0013 | -0.1957 | | -0.0667 | 0.9784 |  |
| 98 | 143.01 | | 39.75 | | 29.5 | | 0.0557 | | 0.0035 | 0.0037 | -0.1957 | | -0.0667 | 0.9784 |  |
| 99 | 143.03 | | 39.99 | | 29.5 | | 0.1062 | | -0.0024 | -0.0006 | -0.1957 | | -0.0667 | 0.9784 |  |
| 100 | 143.05 | | 40.22 | | 29.5 | | 0.0909 | | 0.0031 | -0.0067 | -0.1957 | | -0.0667 | 0.9784 |  |
| 101 | 140.74 | | 35.61 | | 34.5 | | 0.0608 | | -0.0191 | 0.0051 | -0.1961 | | 0.0065 | 0.9806 |  |
| 102 | 140.88 | | 35.85 | | 34.5 | | 0.0813 | | -0.0249 | -0.0054 | -0.1961 | | 0.0065 | 0.9806 |  |
| 103 | 141.02 | | 36.10 | | 34.5 | | 0.0515 | | -0.0232 | -0.0269 | -0.1961 | | 0.0065 | 0.9806 |  |
| 104 | 141.16 | | 36.34 | | 34.5 | | 0.0019 | | -0.0225 | -0.0356 | -0.1961 | | 0.0065 | 0.9806 |  |
| 105 | 141.34 | | 36.58 | | 34.5 | | -0.0270 | | -0.0176 | -0.0259 | -0.1960 | | 0.0382 | 0.9799 |  |
| 106 | 141.54 | | 36.82 | | 34.5 | | -0.0236 | | -0.0110 | -0.0215 | -0.1960 | | 0.0382 | 0.9799 |  |
| 107 | 141.74 | | 37.06 | | 34.5 | | -0.0004 | | -0.0110 | -0.0285 | -0.1960 | | 0.0382 | 0.9799 |  |
| 108 | 141.94 | | 37.29 | | 34.5 | | 0.0181 | | -0.0209 | -0.0467 | -0.1960 | | 0.0382 | 0.9799 |  |
| 109 | 142.11 | | 37.55 | | 34.5 | | 0.0290 | | -0.0339 | -0.0639 | -0.1961 | | -0.0089 | 0.9805 |  |
| 110 | 142.24 | | 37.83 | | 34.5 | | 0.0447 | | -0.0348 | -0.0701 | -0.1961 | | -0.0089 | 0.9805 |  |
| 111 | 142.37 | | 38.10 | | 34.5 | | 0.0664 | | -0.0036 | -0.0746 | -0.1961 | | -0.0089 | 0.9805 |  |
| 112 | 142.50 | | 38.38 | | 34.5 | | 0.0754 | | 0.0534 | -0.0782 | -0.1961 | | -0.0089 | 0.9805 |  |
| 113 | 142.58 | | 38.64 | | 34.5 | | 0.0485 | | 0.1167 | -0.0674 | -0.1958 | | -0.0568 | 0.9790 |  |
| 114 | 142.62 | | 38.88 | | 34.5 | | -0.0128 | | 0.1510 | -0.0533 | -0.1958 | | -0.0568 | 0.9790 |  |
| 115 | 142.66 | | 39.12 | | 34.5 | | -0.0781 | | 0.1410 | -0.0395 | -0.1958 | | -0.0568 | 0.9790 |  |
| 116 | 142.69 | | 39.36 | | 34.5 | | -0.1033 | | 0.0930 | -0.0239 | -0.1958 | | -0.0568 | 0.9790 |  |
| 117 | 142.72 | | 39.60 | | 34.5 | | -0.0656 | | 0.0398 | -0.0074 | -0.1957 | | -0.0667 | 0.9784 |  |
| 118 | 142.74 | | 39.84 | | 34.5 | | 0.0136 | | 0.0093 | 0.0000 | -0.1957 | | -0.0667 | 0.9784 |  |
| 119 | 142.77 | | 40.07 | | 34.5 | | 0.0794 | | 0.0052 | -0.0030 | -0.1957 | | -0.0667 | 0.9784 |  |
| 120 | 142.79 | | 40.31 | | 34.5 | | 0.0776 | | 0.0086 | -0.0072 | -0.1957 | | -0.0667 | 0.9784 |  |
| 121 | 140.47 | | 35.70 | | 39.5 | | 0.0446 | | -0.0269 | -0.0115 | -0.1961 | | 0.0065 | 0.9806 |  |
| 122 | 140.62 | | 35.94 | | 39.5 | | 0.0619 | | -0.0407 | -0.0232 | -0.1961 | | 0.0065 | 0.9806 |  |
| 123 | 140.76 | | 36.19 | | 39.5 | | 0.0387 | | -0.0417 | -0.0325 | -0.1961 | | 0.0065 | 0.9806 |  |
| 124 | 140.90 | | 36.43 | | 39.5 | | -0.0049 | | -0.0338 | -0.0233 | -0.1961 | | 0.0065 | 0.9806 |  |
| 125 | 141.07 | | 36.67 | | 39.5 | | -0.0343 | | -0.0084 | -0.0034 | -0.1960 | | 0.0382 | 0.9799 |  |
| 126 | 141.28 | | 36.91 | | 39.5 | | -0.0441 | | 0.0216 | -0.0127 | -0.1960 | | 0.0382 | 0.9799 |  |
| 127 | 141.48 | | 37.14 | | 39.5 | | -0.0489 | | 0.0358 | -0.0533 | -0.1960 | | 0.0382 | 0.9799 |  |
| 128 | 141.68 | | 37.38 | | 39.5 | | -0.0671 | | 0.0218 | -0.1088 | -0.1960 | | 0.0382 | 0.9799 |  |
| 129 | 141.85 | | 37.64 | | 39.5 | | -0.0859 | | -0.0069 | -0.1445 | -0.1961 | | -0.0089 | 0.9805 |  |
| 130 | 141.98 | | 37.91 | | 39.5 | | -0.0812 | | -0.0271 | -0.1486 | -0.1961 | | -0.0089 | 0.9805 |  |
| 131 | 142.11 | | 38.19 | | 39.5 | | -0.0536 | | -0.0068 | -0.1460 | -0.1961 | | -0.0089 | 0.9805 |  |
| 132 | 142.24 | | 38.47 | | 39.5 | | -0.0282 | | 0.0401 | -0.1433 | -0.1961 | | -0.0089 | 0.9805 |  |
| 133 | 142.32 | | 38.72 | | 39.5 | | -0.0339 | | 0.0949 | -0.1174 | -0.1958 | | -0.0568 | 0.9790 |  |
| 134 | 142.36 | | 38.97 | | 39.5 | | -0.0749 | | 0.1226 | -0.0819 | -0.1958 | | -0.0568 | 0.9790 |  |
| 135 | 142.39 | | 39.21 | | 39.5 | | -0.1251 | | 0.1138 | -0.0486 | -0.1958 | | -0.0568 | 0.9790 |  |
| 136 | 142.43 | | 39.45 | | 39.5 | | -0.1424 | | 0.0741 | -0.0206 | -0.1958 | | -0.0568 | 0.9790 |  |
| 137 | 142.46 | | 39.69 | | 39.5 | | -0.1042 | | 0.0335 | -0.0034 | -0.1957 | | -0.0667 | 0.9784 |  |
| 138 | 142.48 | | 39.92 | | 39.5 | | -0.0276 | | 0.0115 | -0.0039 | -0.1957 | | -0.0667 | 0.9784 |  |
| 139 | 142.50 | | 40.16 | | 39.5 | | 0.0402 | | 0.0087 | -0.0127 | -0.1957 | | -0.0667 | 0.9784 |  |
| 140 | 142.53 | | 40.40 | | 39.5 | | 0.0514 | | 0.0101 | -0.0154 | -0.1957 | | -0.0667 | 0.9784 |  |
| 141 | 140.21 | | 35.79 | | 44.5 | | 0.0211 | | -0.0189 | -0.0189 | -0.1961 | | 0.0065 | 0.9806 |  |
| 142 | 140.36 | | 36.03 | | 44.5 | | 0.0296 | | -0.0318 | -0.0272 | -0.1961 | | 0.0065 | 0.9806 |  |
| 143 | 140.50 | | 36.27 | | 44.5 | | 0.0138 | | -0.0347 | -0.0256 | -0.1961 | | 0.0065 | 0.9806 |  |
| 144 | 140.64 | | 36.52 | | 44.5 | | -0.0170 | | -0.0261 | -0.0089 | -0.1961 | | 0.0065 | 0.9806 |  |
| 145 | 140.81 | | 36.76 | | 44.5 | | -0.0373 | | -0.0004 | 0.0117 | -0.1960 | | 0.0382 | 0.9799 |  |
| 146 | 141.02 | | 36.99 | | 44.5 | | -0.0432 | | 0.0317 | 0.0024 | -0.1960 | | 0.0382 | 0.9799 |  |
| 147 | 141.22 | | 37.23 | | 44.5 | | -0.0532 | | 0.0510 | -0.0473 | -0.1960 | | 0.0382 | 0.9799 |  |
| 148 | 141.42 | | 37.47 | | 44.5 | | -0.0806 | | 0.0378 | -0.1142 | -0.1960 | | 0.0382 | 0.9799 |  |
| 149 | 141.59 | | 37.73 | | 44.5 | | -0.1068 | | 0.0067 | -0.1521 | -0.1961 | | -0.0089 | 0.9805 |  |
| 150 | 141.72 | | 38.00 | | 44.5 | | -0.1072 | | -0.0186 | -0.1493 | -0.1961 | | -0.0089 | 0.9805 |  |
| 151 | 141.85 | | 38.28 | | 44.5 | | -0.0838 | | -0.0064 | -0.1412 | -0.1961 | | -0.0089 | 0.9805 |  |
| 152 | 141.98 | | 38.55 | | 44.5 | | -0.0587 | | 0.0215 | -0.1385 | -0.1961 | | -0.0089 | 0.9805 |  |
| 153 | 142.06 | | 38.81 | | 44.5 | | -0.0535 | | 0.0524 | -0.1087 | -0.1958 | | -0.0568 | 0.9790 |  |
| 154 | 142.10 | | 39.05 | | 44.5 | | -0.0738 | | 0.0667 | -0.0663 | -0.1958 | | -0.0568 | 0.9790 |  |
| 155 | 142.13 | | 39.29 | | 44.5 | | -0.1037 | | 0.0619 | -0.0289 | -0.1958 | | -0.0568 | 0.9790 |  |
| 156 | 142.17 | | 39.54 | | 44.5 | | -0.1159 | | 0.0405 | -0.0037 | -0.1958 | | -0.0568 | 0.9790 |  |
| 157 | 142.20 | | 39.77 | | 44.5 | | -0.0931 | | 0.0201 | 0.0014 | -0.1957 | | -0.0667 | 0.9784 |  |
| 158 | 142.22 | | 40.01 | | 44.5 | | -0.0425 | | 0.0078 | -0.0109 | -0.1957 | | -0.0667 | 0.9784 |  |
| 159 | 142.24 | | 40.25 | | 44.5 | | 0.0062 | | 0.0051 | -0.0233 | -0.1957 | | -0.0667 | 0.9784 |  |
| 160 | 142.26 | | 40.49 | | 44.5 | | 0.0216 | | 0.0053 | -0.0229 | -0.1957 | | -0.0667 | 0.9784 |  |
|  |  |  |  |  |  |  |  |  |  |  |  |  |  |  |  |
| Subf. | Lon | | Lat | | Depth | | Stress vector | | | |  | |  |  |  |
|  |  |  |  |  |  |  | T*x* | | T*y* | T*z* |  | |  |  |  |
| # | (°) | | (°) | | (km) | | （MPa） | | （MPa） | （MPa） |  | |  |  |  |
| B1 | 141.00 | | 33.00 | | 27 | | 0.0065 | | -0.0157 | -0.0005 |  | |  |  |  |
| B2 | 140.90 | | 34.08 | | 27 | | 0.0050 | | -0.0254 | 0.0003 |  | |  |  |  |
| B3 | 140.95 | | 34.94 | | 27 | | -0.0104 | | -0.0127 | 0.0009 |  | |  |  |  |
| B4 | 143.37 | | 40.69 | | 27 | | -0.0518 | | -0.0162 | 0.0026 |  | |  |  |  |
| B5 | 143.78 | | 41.35 | | 27 | | -0.0013 | | 0.0154 | 0.0010 |  | |  |  |  |
| B6 | 144.51 | | 41.87 | | 27 | | 0.0305 | | 0.0046 | -0.0010 |  | |  |  |  |
| B7 | 145.43 | | 42.34 | | 27 | | 0.0314 | | -0.0023 | -0.0016 |  | |  |  |  |
| B8 | 138.57 | | 33.82 | | 123.5 | | 0.0093 | | -0.0244 | -0.0072 |  | |  |  |  |
| B9 | 138.46 | | 34.90 | | 123.5 | | 0.0226 | | -0.0195 | -0.0052 |  | |  |  |  |
| B10 | 138.52 | | 35.76 | | 123.5 | | 0.0211 | | 0.0098 | 0.0123 |  | |  |  |  |
| B11 | 138.91 | | 36.66 | | 123.5 | | -0.0033 | | 0.0147 | -0.0026 |  | |  |  |  |
| B12 | 139.60 | | 37.62 | | 123.5 | | 0.0451 | | 0.0142 | -0.0018 |  | |  |  |  |
| B13 | 140.26 | | 38.65 | | 123.5 | | 0.0868 | | -0.0180 | 0.0223 |  | |  |  |  |
| B14 | 140.60 | | 39.68 | | 123.5 | | 0.0513 | | 0.0416 | 0.0099 |  | |  |  |  |
| B15 | 140.71 | | 40.64 | | 123.5 | | -0.0547 | | -0.0147 | -0.0192 |  | |  |  |  |
| B16 | 140.94 | | 41.51 | | 123.5 | | -0.0266 | | 0.0059 | 0.0164 |  | |  |  |  |
| B17 | 141.35 | | 42.17 | | 123.5 | | 0.0571 | | 0.0412 | 0.0017 |  | |  |  |  |
| B18 | 142.07 | | 42.69 | | 123.5 | | 0.1074 | | 0.0499 | -0.0140 |  | |  |  |  |
| B19 | 143.00 | | 43.15 | | 123.5 | | 0.0923 | | 0.0253 | -0.0072 |  | |  |  |  |
